# Novel symbionts and potential human pathogens excavated from argasid tick microbiomes that are shaped by dual or single symbiosis

**DOI:** 10.1016/j.csbj.2022.04.020

**Published:** 2022-04-19

**Authors:** Mohamed Abdallah Mohamed Moustafa, Wessam Mohamed Ahmed Mohamed, Alice C.C. Lau, Elisha Chatanga, Yongjin Qiu, Naoki Hayashi, Doaa Naguib, Kozue Sato, Ai Takano, Keita Matsuno, Nariaki Nonaka, DeMar Taylor, Hiroki Kawabata, Ryo Nakao

**Affiliations:** a Laboratory of Parasitology, Department of Disease Control, Faculty of Veterinary Medicine, Hokkaido University, Sapporo, Hokkaido 060-0818, Japan; b Department of Animal Medicine, Faculty of Veterinary Medicine, South Valley University, Qena 83523, Egypt; c Division of Bioinformatics, Research Center for Zoonosis Control, Hokkaido University, Sapporo, Hokkaido 001-0020, Japan; d Laboratory of Wildlife Biology and Medicine, Department of Environmental Veterinary Sciences, Faculty of Veterinary Medicine, Hokkaido University, Sapporo, Hokkaido 060-0818, Japan; e Department of Veterinary Pathobiology, Faculty of Veterinary Medicine, Lilongwe University of Agriculture and Natural Resources, P.O. Box 219, Lilongwe, Malawi; f Division of International Research Promotion, International Institute for Zoonosis Control, Hokkaido University, Sapporo, Hokkaido 001-0020, Japan; g Department of Hygiene and Zoonoses, Faculty of Veterinary Medicine, Mansoura University, Mansoura 35516, Egypt; h Laboratory of Systemic Infection, Department of Bacteriology-I National Institute of Infectious Diseases Toyama 1-23-1, Shinjuku-ku, Tokyo 162-8640 Japan; i Laboratory of Epidemiology, Department of Veterinary Medicine, Joint Faculty of Veterinary Medicine, Yamaguchi University, 1677-1 Yoshida, Yamaguchi 753-8515, Japan; j Division of Risk Analysis and Management, International Institute for Zoonosis Control, Hokkaido University, Sapporo, Hokkaido 001-0020, Japan; k Faculty of Life and Environmental Sciences, University of Tsukuba, Tsukuba, Ibaraki 305-8572, Japan; l Department of Bacteriology-I, National Institute of Infectious Diseases, Toyama 1-23-1, Shinjuku-ku, Tokyo 162-8640, Japan; m One Health Research Center, Hokkaido University, Sapporo, Hokkaido 001-0020, Japan; n International Collaboration Unit, International Institute for Zoonosis Control, Hokkaido University, Sapporo, Hokkaido 001-0020, Japan

**Keywords:** Argasid ticks, Dual symbiosis, Infection, Pathogens, Vector-borne diseases

## Abstract

Research on vector-associated microbiomes has been expanding due to increasing emergence of vector-borne pathogens and awareness of the importance of symbionts in the vector physiology. However, little is known about microbiomes of argasid (or soft-bodied) ticks due to limited access to specimens. We collected four argasid species (*Argas japonicus*, *Carios vespertilionis*, *Ornithodoros capensis*, and *Ornithodoros sawaii*) from the nests or burrows of their vertebrate hosts. One laboratory-reared argasid species (*Ornithodoros moubata)* was also included. Attempts were then made to isolate and characterize potential symbionts/pathogens using arthropod cell lines. Microbial community structure was distinct for each tick species. *Coxiella* was detected as the predominant symbiont in four tick species where dual symbiosis between *Coxiella* and *Rickettsia* or *Coxiella* and *Francisella* was observed in *C. vespertilionis* and *O. moubata*, respectively. Of note, *A. japonicus* lacked *Coxiella* and instead had *Occidentia massiliensis* and Thiotrichales as alternative symbionts. Our study found strong correlation between tick species and life stage. We successfully isolated *Oc. massiliensis* and characterized potential pathogens of genera *Ehrlichia* and *Borrelia*. The results suggest that there is no consistent trend of microbiomes in relation to tick life stage that fit all tick species and that the final interpretation should be related to the balance between environmental bacterial exposure and endosymbiont ecology. Nevertheless, our findings provide insights on the ecology of tick microbiomes and basis for future investigations on the capacity of argasid ticks to carry novel pathogens with public health importance.

## Graphical abstract

**Figure:**
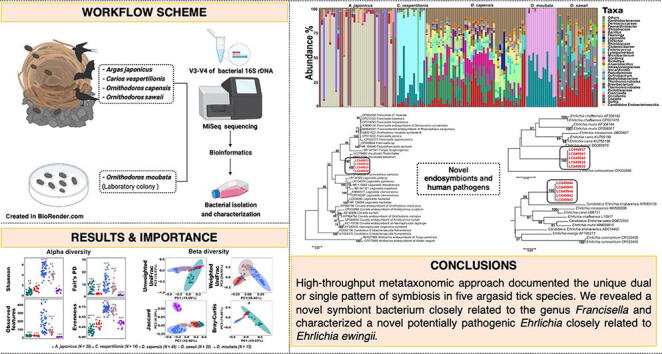

## 1 Introduction

Ticks are obligatory blood-feeding arachnids and the second major vector transmitting pathogens to humans and animals [1]. Studies have shown that ticks pose a threat to human and animals not only through transmitting a wide range of pathogens but also by causing blood loss, allergic reactions, and paralysis [2], [3], [4]. There are three tick families: Ixodidae (ixodid or hard-bodied ticks), Argasidae (argasid or soft-bodied ticks), and Nuttalliellidae (only one species) [5]. While each stage of female ixodid ticks feed only once and may have a diverse habitat, male ixodid ticks may feed several times. Some stages of argasids feed for a few hours or not at all (for example, *Ornithodoros moubata* larvae molt into nymphs without blood meals) [6] and live in hidden sites near their hosts [7]. More than 900 tick species have been reported [8] with more than 186 species classified as argasid species [9].

Generally, ticks harbor a wide variety of microbes, including endosymbionts, commensals, and tick-borne pathogens (TBPs) [10] that represent a complex microbiome. Some endosymbionts such as *Coxiella*, *Francisella*, and *Rickettsia* species are essential for ticks to provide the nutrients lacking in their blood meals [11]. In most cases, these symbionts are maternally inherited and have been associated with their vector hosts for millions of years [12]. Recently, studies describe more than seven endosymbiont bacterial genera from several tick species [13]. In addition, ticks harbor a wide variety of commensal bacteria from the surrounding environment and the host skin [14] such as *Bacillus*, *Mycobacterium*, *Methylobacterium*, and *Pseudomonas* [15]. Ticks are also frequently co-infected with several TBPs that can cause diseases in humans and animals [16]. Although many studies have described the microbiome of several tick species [17], [18], [19], only few have discussed the complexity of the epidemiology and network of interactions of these microbes within ticks [20], [21], [22], [23], [24]. For example, significant ecological associations have been detected between the nonpathogenic microbes and pathogens in some ixodid ticks [25], [26]. To solve this complex network, an integration of metataxonomic approaches, bioinformatics and experimental design is required. Hence, an investigation on the rarely described microbiome and microbial interactions in argasid ticks is timely required to understand the role they play in the transmission of pathogens.

The global number of newly described argasid ticks has been increasing [27], [28], [29]. These argasid ticks have a high opportunity to acquire and transmit pathogens to their hosts in each developmental stage [14]. For instance, argasid ticks harbor several *Rickettsia* spp. [17], [30], [31], [32], *Borrelia* spp. [33], [34], [35], and *Ehrlichia* spp. [36]. To date, most of the large-scale studies on tick microbiomes have focused only on ixodid ticks [37] and little has been discussed about argasid ticks. Unlike ixodid ticks, ticks of the family Argasidae are nidicolous, having long life spans (6–9 years), and more frequent and fast feeders (several minutes to hours) [38], [39], [40]. Sequentially, all life stages of these ticks could coexist in one habitat, which can lead to maintaining several bacterial species throughout the developmental stages and sexes [41].

In Japan, a total of four argasid ticks have been described as *Argas japonicus*, *Carios vespertilionis*, *Ornithodoros capensis*, and *Ornithodoros sawaii* [42]. However, little is known about the role of these ticks in the transmission of TBPs to both humans and animals in a country where many human cases of tick-borne diseases (TBDs) are reported [43]. In addition, characterization of the diversity and composition of microbes associated with several argasid tick species is required to understand the evolutionary, ecological, and physiological functions of the tick-associated microbiome [44]. In addition, the study of tick microbial communities can help to improve the control strategies of ticks and TBPs [45] and identify novel bacterial species that may be pathogenic to humans and animals [46]. Recently, a study described the microbial diversity in *A. japonicus* samples collected from China [17], but the dominant endosymbiont bacteria was not clearly identified in these samples. In Mexico, two bacteria (*Midichloria* and *Coxiella*) were suggested to be the endosymbionts of *O. turicata* [47] based on the analysis of 17 adult ticks collected from the Bolson tortoise (*Gopherus flavomarginatus*). Similarly, the microbiomes of seabird ticks (*O. maritimus* and *O. capensis*) were found to be dominated by *Coxiella* endosymbionts [41], [48]. However, the microbiome of *O. moubata*, which is considered the most studied among argasid ticks, is dominated by two endosymbionts, *Coxiella* and *Francisella* [49], [50].

In this study, four argasid tick species, *A. japonicus*, *C. vespertilionis*, *O. capensis*, and *O. sawaii* were used to characterize the microbiome composition and diversity in argasid ticks from Japan. To understand the relationship between the tick microbiome and the surrounding environment, we investigated the microbiome of the African *O. moubata* from a laboratory colony maintained at the University of Tsukuba, Japan. We report isolation and genetic characterization of a potentially novel endosymbiont detected in *A. japonicus.* Further, we targeted flagellin protein gene (*flaB*), citrate synthase gene (*gltA*), and chaperonin gene (*groEL*) to characterize a *Borrelia* and a potential novel *Ehrlichia* species that we detected in our samples. Collectively, our study can help to better understand the interrelationships between argasid ticks, their associated microbiomes, and the identification of potentially pathogenic bacteria.

## 2 Materials and methods

### 2.1 Argasid tick samples and DNA extraction

A total of 122 argasid ticks from four species, *A. japonicus* (*n* = 38; 14 males, 20 females, and 4 nymphs), *C. vespertilionis* (*n* = 14; 6 females and 8 nymphs), *O. capensis* (*n* = 49; 18 males, 14 females, and 17 nymphs), and *O. sawaii* (*n* = 21; 8 males, 6 females, and 7 nymphs) were collected from Yamanashi, Nagano, Kagoshima, and Kyoto Prefectures, Japan, respectively. Briefly, *A. japonicus* and *C. vespertilionis* ticks were collected from nests of Red-rumped swallow (*Hirundo daurica*) and Japanese short-tailed bat (*Eptesicus japonensis*), respectively*.* In addition, *O. capensis* and *O. sawaii* ticks were collected from soils in the burrows of Swinhoe's strom petrel (*Oceanodroma monorhis*) and Streaked Shearwaters (*Calonectris leucomelas*), respectively. They were morphologically identified based on a standard key under a stereomicroscope [51], [52]. In addition, a total of 15 *O. moubata* (5 males, 5 females, and 5 nymphs) were obtained from a laboratory colony maintained at the University of Tsukuba, Japan for more than 20 years.

All ticks were processed alive under sterile conditions. Tick DNA was extracted as described elsewhere [53]. In brief, the tick surface was washed once with 70% ethanol and twice with sterile PBS. After homogenizing in 100 μL of high glucose Dulbecco’s Modified Eagle Medium (DMEM) (Gibco, Life Technologies, Gland Island, NY, USA) using 4.8 mm stainless beads in a Micro Smash MS-100R (TOMY, Tokyo, Japan), DNA was extracted from 50 μL of the tick homogenate using a blackPREP Tick DNA/RNA Kit (Analytikjena, Jena, Germany) according to the manufacturer’s instructions. The remaining homogenates were kept at − 80 °C for future use in bacterial isolation. Detailed information about the collected tick samples (tick species, collection site, and sex/stage) are provided in Table S1.

### 2.2 MiSeq 16S rRNA gene amplicon sequencing

A total of 137 argasid tick genomic DNA samples, two DNA extraction blank controls and six distilled water PCR negative controls were used for PCR reactions to amplify the V3-V4 regions of the bacterial 16S rRNA gene using Illumina barcoded primers. Briefly, the primer Illumina_16S_341F and Illumina_16S_805R was used to amplify the V3-V4 region of the 16S rRNA gene by PCR [54], [55] using Kapa HiFi HotStart Ready Mix (KAPA Biosystems, Wilmington, MA, USA). Each PCR reaction consisted of 12.5 μL of 2 × KAPA HiFi HotStart ReadyMix, 5.0 μL of each primer and 2.5 μL of the tick genomic DNA samples or negative controls. PCR results were confirmed by electrophoresis using 1.5% agarose gel stained with Gel-Red^TM^ (Biotium, Hayward, CA, USA) and visualized under UV light. The amplicons were purified using AMPure XP (Beckman Coulter Life Sciences, IN, USA). A library was prepared using the Nextera Index Kit (Illumina, San Diego, CA, USA) and sequenced with a MiSeq Reagent Kit v3 (600 cycles) on an Illumina MiSeq device according to the manufacturer’s instructions. The raw sequence data were deposited in the DNA Data Bank of the Japan Sequence Read Archive under the DRA accession number: DRA013238.

### 2.3 Bioinformatics processing

We used the Quantitative Insights Into Microbial Ecology 2 Software (QIIME2) (version 2020.2) [56] as a platform for microbiome bioinformatics in argasid ticks. The raw sequencing data were obtained from BaseSpace (Illumina), and then demultiplexed, quality-checked, and filtered using q2‐demux plugin followed by denoising with DADA2 pipeline (version: 2019.10) [57]. The obtained amplicon sequence variants (ASVs) were aligned using q2‐alignment plugin by mafft [58]. A phylogeny was constructed using q2‐phylogeny plugin by fasttree2 [59]. We selected a sampling depth of 7,760 reads for comparing the diversity analysis using q2‐diversity plugin in QIIME2 between the examined argasid tick species and excluded one sample from *O. sawaii* due to low number of reads. In addition, we compared the diversity values according to sex and stage within each argasid tick species using a sampling depth of 27342, 9442, 17,878, 14,069, and 7,760 for *A. japonicus*, *C. vespertilionis*, *O. capensis*, *O. sawaii*, and *O. moubata*, respectively. We calculated alpha diversity based on Shannon diversity [60] (accounts for richness and evenness), Faith’s Phylogenetic Diversity (Faith’s PD) [61] (accounts for the branch lengths of the rooted phylogenetic tree connecting the detected bacterial species), observed features [62] (accounts for the number of taxonomic groups), and Pielou’s evenness [63] (accounts for the abundance of species relative to other species in a given community). We exported and visualized the results in R using the qiime2R, ggplot2, and phyloseq packages [64]. In addition, beta diversity was calculated based on unweighted UniFrac distance [65] (presence or absence of observed species), weighted UniFrac distance [66] (abundance of observed species), Jaccard similarity index [67] (the number of shared species divided by the total number of species detected in the sample sets), and Bray-Curtis dissimilarity [68] (differences between sites in terms of species counts present in those sites) analyses using QIIME2. Furthermore, visualization of clustering of ASVs according to species and sex/stage was performed by a Principal Coordinates Analysis (PCoA) using EMPeror plugin in QIIME2 [69] and R as described above. The taxonomy was assigned using q2‐feature‐classifier plugin [70] with classify‐sklearn naïve Bayes taxonomy classifier and SILVA classifier reference sequences (release 132). The frequency method in the Decontam package [71] in R (version 4.0.2) [72] was implemented with a threshold of 0.1 to identify the likely contaminants introduced during processing. Finally, ASVs identified as archaea, eukaryota, and potential contaminants as well as sequences not assigned to kingdom level were removed manually for further analysis in QIIME2 using sequence identifiers. A heatmap phylogenetic tree was constructed using the heatmap method in QIIME2 [73]. We visualized the differential abundance of the 30 most abundant taxonomic groups using taxa_heatmap function in the qiime2R package in R (version 2.13.0). Finally, we identified the bacteria contributing to the dissimilarity of the microbiome among the tick groups by implementing the linear discriminant analysis effect size (LEfSe) in the Huttenhower lab Galaxy pipeline [74]. LEfSe was implemented to compare among (between different species for the same sex and stage) and within (between different sex and stage for each species) tick species variations.

### 2.4 Statistical analyses

We estimated the statistical differences in alpha diversities among argasid tick species using a generalized linear model (GLM). The response variable was alpha diversity including Shannon diversity calculated as the exponential function of Shannon entropy [75], Faith’s PD, observed features, and Pielou’s evenness with tick species and sex/stage (female, male, and nymph) as fixed effect variables. We checked various model assumptions, including normality of residuals, normality of random effects, linear relationship, homogeneity of variance, and multicollinearity, using the check_model function in the performance package (version 0.8.0) in R [76]. We used the emmeans package in R [77] as a *post hoc* method to show the resulted pairwise comparisons. The modeling was performed using the ﻿stats package version ﻿4.0.3 in R [72]. We then analyzed the effect of species and sex/stage on beta diversity among all argasid ticks using Adonis Permutational multivariate analysis of variance (PERMANOVA) with 999 permutations. Afterwards, we ran a series of pairwise comparisons between all argasid species. We also tested the significance in beta diversity differences within each argasid tick species according to sex/stage using pairwise PERMANOVA [78] with 999 permutations.

### 2.5 Isolation and characterization of potential endosymbionts

A total of 30 tick homogenates (5 *C. vespertilionis*, 12 *A. japonicus*, 6 *O. capensis*, 5 *O. sawaii*, and 2 *O. moubata*) were selected to isolate potential endosymbionts using arthropod cell lines as described previously [53]. In brief, tick homogenates (5 μL each) were inoculated to ISE6 (*Ixodes scapularis* embryos) cell and C6/36 (*Aedes albopictus* larvae) cell cultures seeded in 24-well culture plates. The culture medium was changed every 3 and 7 days for C6/36 and ISE6 cells, respectively. Every 2 weeks, one hundred μL of culture suspension was blindly passaged into new wells containing uninfected cells. The experiment was terminated at 8 weeks post inoculation. When fungal or bacterial contamination was observed, the contaminated wells were sterilized with 10% hypochlorous acid.

In addition to microscopic observations to detect cytopathic effects caused by bacterial infections, crude DNA extracted from 10 μL of culture suspension at 4- and 8-weeks post inoculation using heat alkali treatment was tested for bacterial infection by universal PCR amplifying bacterial 16S rRNA gene. PCR was carried out using the high-fidelity KOD-Plus-Neo DNA polymerase (Toyobo, Osaka, Japan) in a 25.0 µL reaction mixture containing 2.5 µL of 10x Buffer for KOD-Plus-Neo, 300 nM of each fD1 and Rp2 primers [79], 2.5 µL of dNTPs, 1.5 µL of 25 mM MgSO_4_, 0.5 unit of KOD-Plus-Neo DNA polymerase, 2.0 µL of template DNA and molecular grade water was used to adjust the volume. The reaction conditions were set at 94 °C for 2 min and 30 cycles of 98 °C for 10 sec, 55 °C for 30 sec and 68 °C for 30 sec, and final extension at 68 °C for 2 min (Table 1).

**Table 1:** Primers used in this study.

Organism	Target gene	Sequence 5′→ 3′	Primer name	Expected size (bp)	Annealing (°C)
Bacteria	16S rRNA	TCGTCGGCAGCGTCAGATGTGTATAAGAGACAGCCTACGGGNGGCWGCAG	Illumina_16S_341F	460	55
		GTCTCGTGGGCTCGGAGATGTGTATAAGAGACAGGACTACHVGGGTATCTAATCC	Illumina_16S_805R		
		AGAGTTTGATCCTGGCTCAG	fD1	1,600	55
		ACGGCTACCTTGTTACGACTT	rP2		
*Ehrlichia* spp.	*gltA*	CAGGATTTATGTCTACTGCTGCTTG	EHRCS-131F (1st)	473	50
		CCAGTATATAAYTGACGWGGACG	EHRCS-1226R (1st)		
		ATGCTGATCATGARCAAAATG	EHRCS-754F (2nd)		50
		CCAGTATATAAYTGACGWGGACG	EHRCS-1226R (2nd)		
	*groEL*	TGGCAAATGTAGTTGTAACAGG	groEL_fwd3	1,100	50
		GCCGACTTTTAGTACAGCAA	groeEL_rev2		
*Borrelia* spp.	*flaB*	GATCARGCWCAAYATAACCAWATGCA	BflaPAD (1st)	345 by 1st, applox. 300 by 2nd	55
		AGATTCAAGTCTGTTTTGGAAAGC	BflaPDU (1st)		
		GCTGAAGAGCTTGGAATGCAACC	BflaPBU (2nd)		50
		TGATCAGTTATCATTCTAATAGCA	BflaPCR (2nd)		
*Wolbachia* sp.	16S rRNA	GGTACCYACAGAAGAAGTCC	EHR16SD	1,395	55
		ACGGYTACCTTGTTACGACTT	1513R		

### 2.6 Characterization of *Ehrlichia*, *Borrelia*, and *Wolbachia*

Characterization of *Ehrlichia* spp. was performed by amplifying approximately 473 bp and 1,100 bp segments of *gltA* and *groEL* genes, respectively, as previously described [80]. Each PCR reaction consisted of 2.0 μL 10 × Ex Taq Buffer, 1.6 μL dNTP Mixture (2.5 mM), 14.5 μL molecular grade water, 0.1 μL TaKaRa Ex Taq HS (5 U/μL) (Takara Bio Inc., Shiga, Japan), 0.4 μL of each primer (10 μM) and 1 μL of the DNA template.

*Borrelia*-positive argasid tick samples were examined by nested PCR targeting a 345 bp segment of *Borrelia* spp. *flaB* using the primer pairs BflaPAD and BflaPDU for the first PCR and BflaPBU and BflaPCR (Table 1) for the secondary PCR [81]. PCR cycling conditions were implemented as previously described [82].

Finally, we examined one *Wolbachia*-positive argasid tick sample by PCR targeting a 1,395 bp segment of *Wolbachia* spp. 16S rRNA gene using the primer pair EHR16SD [83] and 1513R [79] (Table 1). The PCR products were electrophoresed in a 1.5% agarose gel stained with Gel-Red and visualized under UV light.

### 2.7 Sanger sequencing and phylogenetic analysis

The resulting PCR products were purified using the NucleoSpin Gel and PCR Clean Up Kit (Takara Bio Inc.) or ExoSAP-IT PCR Product Cleanup Reagent (Thermo Fisher Scientific, Waltham, MA, USA) and sequenced by using the BigDye Terminator v3.1 Cycle Sequencing Kit (Applied Biosystems, Foster City, CA, USA) and an ABI Prism 3130 × genetic analyser (Applied Biosystems) according to the manufacturers' instructions.

The obtained sequences were imported to Geneious v10.2.6 (Biomatters Ltd., Auckland, New Zealand) and the primer sequences were removed. The assembled sequences were submitted to the DNA Data Bank of Japan (DDBJ) under the accession numbers LC649932-LC649949.

The nucleotide Basic Local Alignment Search Tool (BLASTn) (accessed on 5 June 2021, https://blast.ncbi.nlm.nih.gov/Blast.cgi) was used to compare the sequences in this study with public databases. We used MAFFT v7.450 software [58] to align the sequences with the previously published related ones. The best fit model for the phylogenetic analysis was estimated using MEGA X software [84] and the trees were built using PHYML v3.3 software [85] with the branches supported by Bootstrap tests.

## 3 Results

### 3.1 Laboratory colonies of *Ornithodoros moubata* harbor less diverse microbiome than field collected argasid ticks

A total of 8,394,800 raw paired-end reads were generated by the Illumina MiSeq from a total of 137 argasid tick and eight negative control samples. The reads were truncated and trimmed according to the demultiplexing summary. The DADA2 quality control analysis and Decontam package output resulted in 4,536,908 high quality paired-end reads classified into 4,279 features. The maximum and minimum frequencies per sample were 61,825 and 4,260, respectively. The lowest total number of reads and features was detected from *O. moubata* (Table 2, Fig. S1).

**Table 2:** Summary of the number of reads for each argasid tick species.

Species	Total reads	Total features	Minimum reads per sample	Maximum reads per sample	Mean reads per sample
*A. japonicus*	1,636,358	237	27,342	61,825	43,062
*C. vespertilionis*	478,921	124	9,442	47,206	34,208
*O. capensis*	1,635,302	3,384	17,878	49,670	33,373
*O. sawaii*	571,346	802	4,260	38,784	27,206
*O. moubata*	214,981	43	7,760	21,924	14,332

### 3.2 Tick species and sex/stage elucidate variations in the associated microbiome diversity

**Fig. 1:**
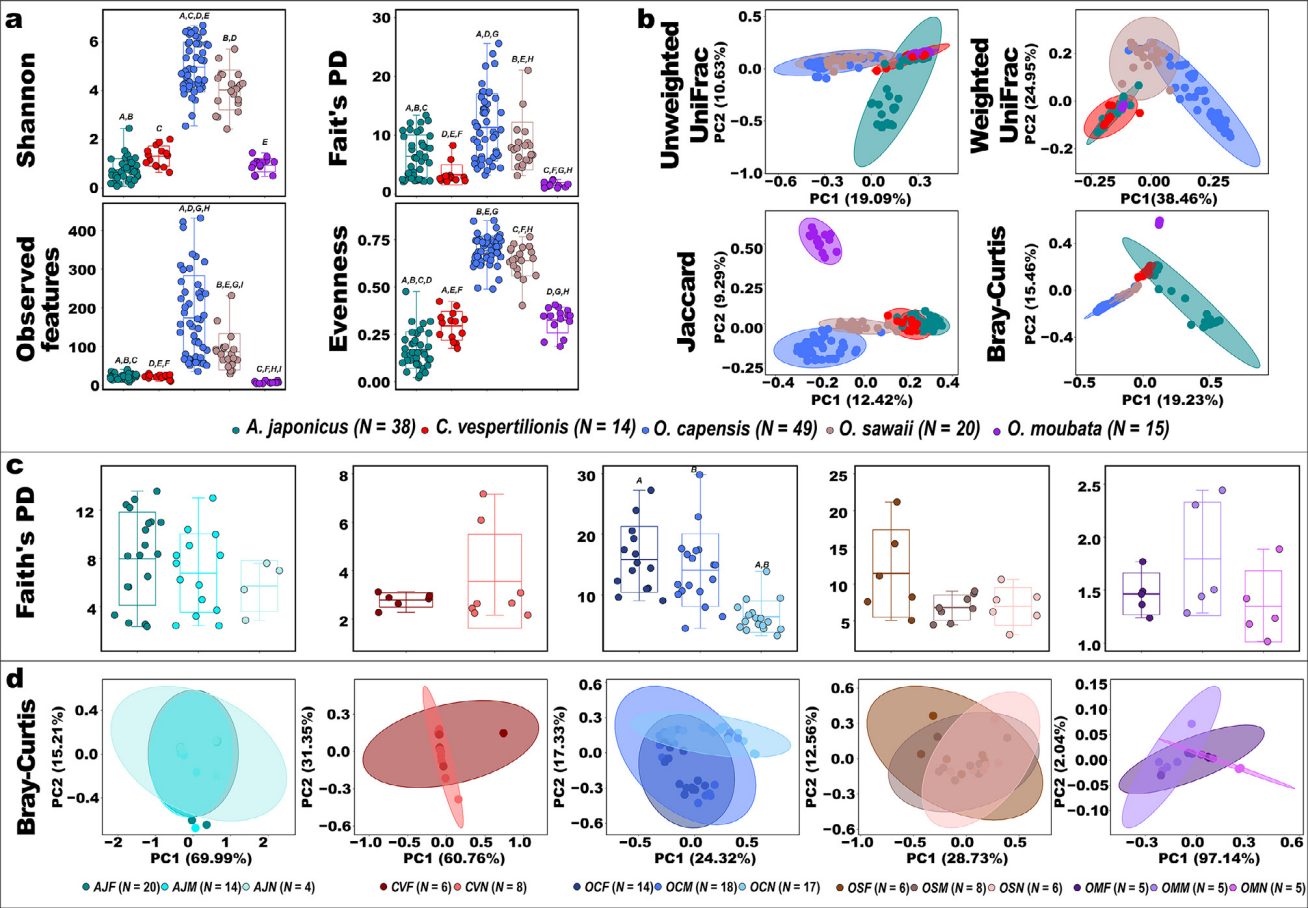
Diversity analyses of microbial populations from 136 argasid tick samples. Each dot shows the microbial population from an individual argasid tick and color represents sample species. Color represents sample species (*A. japonicus* “*AJ*”, *C. vespertilionis* “*AV*”, *O. capensis* “*OC*”, *O. sawaii* “*OS*”, and *O. moubata* “*OM*”), sex (Female “*F*” and Male “*M*” or stage (Nymph “*N*”). a) Alpha diversity analyses according to species variation. Argasid tick species predicted significant variations in the four-alpha diversity metrices (GLM: *p* < 0.001). b) Beta diversity analyses among argasid ticks. c) Faith's phylogenetic diversity analysis according to sex and stage variations. d) PCoA plots based on Bray-Curtis dissimilarity between individual argasid samples according to sex and stage variation. Same Letters above the bars indicate statistically significant difference (GLM: *p* < 0.01).

There were no potential issues in our GLMs based on the visualized values of model assumptions that were used to test significance in alpha diversity among the tested argasid ticks (Fig. S2-5). The alpha diversity of tick microbiome among the examined argasid ticks using Shannon, Faith’s PD, observed features, and Pielou’s evenness analyses is significantly affected by the tick species variation as tested by GLM (*p* < 0.01) (Fig. 1 and Table S2-5). Interestingly, we found that the four alpha diversity metrices were significantly lower in *Argas* and *Carios* species when compared to *Ornithodoros* spp. collected from the environments (*p* < 0.01) (Fig. 1, Fig. S6, and Table S2-5) except that Shannon diversity results of *A. japonicus* and *C. vespertilionis* were not significantly different from *O. moubata* and *O. sawii*, respectively. In addition, Pielou’s evenness of *C. vespertilionis* was not significantly different from *O. moubata*. We also found that all alpha diversity metrices, except the observed features, of *O. capensis* were not significantly different from *O. sawaii*. The analysis also showed significant differences in Shannon diversity, Faith’s PD, and the observed features of adult argasid ticks when compared to nymphs.

Four alpha diversity metrices showed non-significant correlation with the sex and stage variations within all examined argasid species except for *O. capensis* where the values in nymphs were significantly lower than in adult stages (Table S6, Fig. 1, and Fig. S6). The estimated Pielou’s evenness diversity within *O. moubata*, the only laboratory colony in this study, were significantly lower in the nymphal stage than in the adult females and males (*p* ≤ 0.01) (Table S6 and Fig. S6). In addition, adonis PERMANOVA showed that the calculated beta diversity metrices including the unweighted UniFrac, weighted UniFrac, Jaccard, and Bray-Curtis analyses were significantly variable according to argasid species and sex/stage (*p* < 0.01) (Table S7). Pairwise PERMANOVA showed that the four beta diversity metrices were significantly different between all argasid species (Table S8 and Fig. S7) and the effect of sex/stage on beta diversity within each species was not consistent as explained among different species. Specifically, only Jaccard was significantly affected by sex and stage variation in *A. japonicus* (*p* < 0.01) (Table S9) and *C. vespertilionis* (*p* < 0.05) (Table S10), respectively. The pairwise analysis of *O. capensis* showed significant differences in all dissimilarity indices in nymphs when compared to females and males (*p* < 0.01) (Table S11). While no significant differences in beta diversity were detected among different sex or stage in *O. sawaii* (*p* > 0.05) (Table S12), weighted UniFrac and Bray-Curtis dissimilarity indices in *O. moubata* were significantly different in nymphs compared to females and males (*p* = 0.04 and *P* = 0.01, respectively) (Table S13)*.*

Our PCoA plots confirmed the pairwise PERMANOVA results as samples from each argasid species clustered together separate from the others (Fig. 1). In addition, there was an overlap in the clustering of sex and stage within species (Fig. 1 and Fig. S8).

### 3.3 Dual symbiosis in argasid ticks and the detection of *novel endosymbionts in Argas japonicus*

**Fig. 2:**
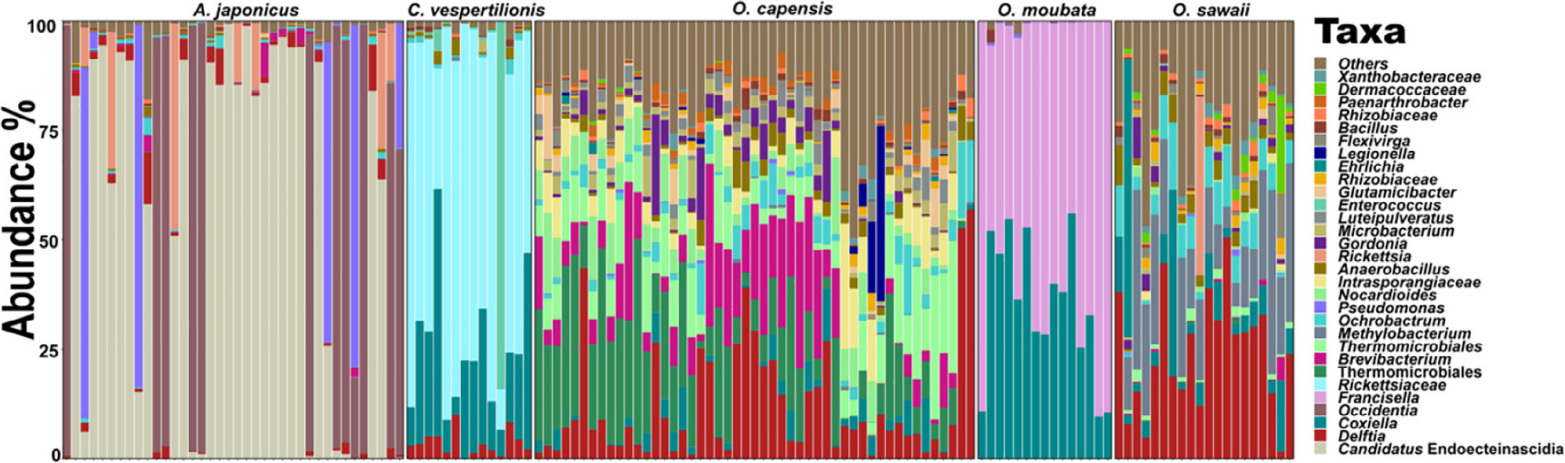
Relative abundance (%) of bacterial taxa identified in the microbiome of five species of argasid ticks. The figure displays the most abundant 30 taxa individually with the remaining grouped together. Each bar represents the bacterial taxa detected in one sample.

The most abundant four bacterial families were completely different among the examined argasid tick species (Table 3 and Fig. S9). We presented the total number of reads for each bacterial feature detected in this study in Table S14. According to the list of the most common maternally inherited bacteria in ticks [44], the microbiome of each examined argasid species included at least one potential endosymbiont and three species (*A. japonicus, C. vespertilionis*, and *O. moubata*) had two potential endosymbionts (Fig. 2 and Fig. S10-14).

**Table 3:** Summary of the most abundant bacterial families in the microbiome of five argasid species.

Tick species	Bacterial family	Abundance (%)
*A. japonicus*	Piscirickettsiaceae*	52.03
	Rickettsiaceae*	33.37
	*Pseudomonadaceae*	8.05
	*Burkholderiaceae*	2.39
*C. vespertilionis*	Rickettsiaceae*	62.51
	Coxiellaceae*	21.21
	*Enterococcaceae*	8.37
	*Burkholderiaceae*	3.88
*O. capensis*	Thermomicrobiales, JG30-KF-CM45	25.47
	*Burkholderiaceae*	13.75
	*Brevibacteriaceae*	10.31
	*Nocardioidaceae*	6.47
*O. sawaii*	*Burkholderiaceae*	26.32
	*Beijerinckiaceae*	20.16
	*Rhizobiaceae*	9.25
	Coxiellaceae*	6.19
*O. moubata*	Francisellaceae*	64.87
	Coxiellaceae*	34.35
	*Enterobacteriaceae*	0.29
	*Pseudomonadaceae*	0.18

* These bacterial families are the potential endosymbionts within each argasid species.

**Fig. 3:**
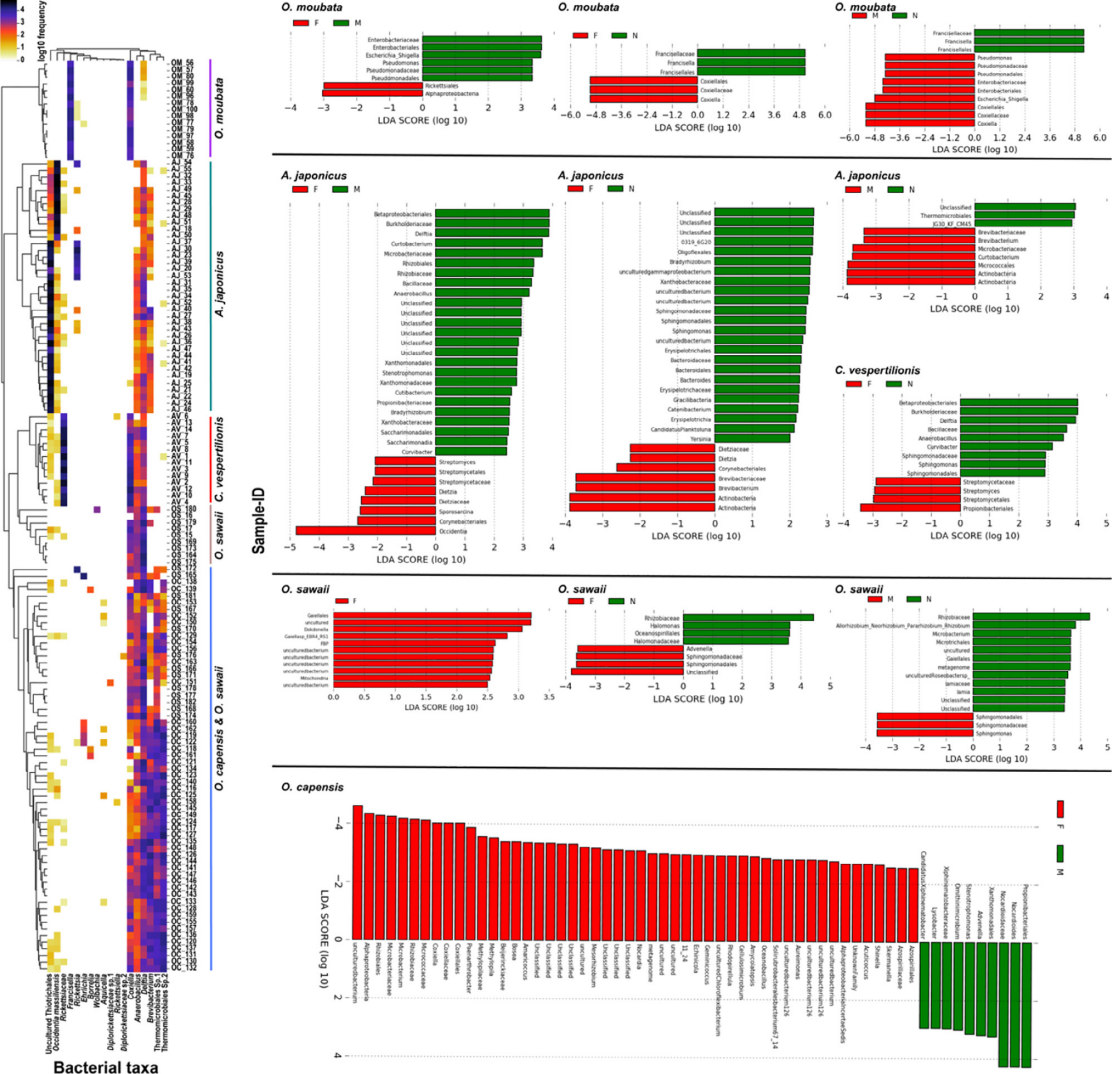
Relative abundance (%) of the potential pathogenic or endosymbiotic bacterial taxa identified in the microbiome of five species of argasid ticks. Sample-ID labels show the argasid tick species (*A. japonicus* “**AJ**”, *C. vespertilionis* “**CV**”, *O. capensis* “**OC**”, *O. sawaii* “**OS**”, and *O. moubata* “**OM**”) and sample numbers. On the right side of the figure, LEfSe results showing the most differentially abundant taxa (*p* < 0.05) within each argasid species according to sex and stage variations.

The relative abundance and LEfSe analyses of the bacterial genera showed a unique presentation of the potential endosymbionts, commensals, and pathogens in the examined five argasid species. A bacterial genus belonging to the family *Piscirickettsiaceae* (identified as *Candidatus* Endoecteinascidia in SILVA classifier) and *Occidentia* species were the most abundant in *A. japonicus* (mean relative abundance = 53.0% and 27.2%, respectively) (Table 4). However, the taxa bar plot showed a negative correlation between both species in *A. japonicus* (Fig. 2 and Fig. S10). In addition, *A. japonicus* samples were divided into two clusters according to the relative abundance of the potential pathogenic or endosymbiotic bacterial taxa, where each cluster was dominated by either *Candidatus* Endoecteinascidia or *Occidentia* species (Fig. 3). The most abundant bacterial genera in *C. vespertilionis* were *Rickettsia* and *Coxiella* (mean relative abundance = 64.0% and 21.4%, respectively) (Table 4, Fig. 3, and Fig. S11).

**Table 4:** Summary of the potential endosymbionts and their relative abundances in the microbiome of five argasid species.

Tick species	Potential endosymbiont	Abundance (%)
*A. japonicus*	Thiotrichales	53.0
	*Occidentia massiliensis*	27.2
*C. vespertilionis*	*Rickettsia*	64.0
	*Coxiella*	21.4
*O. capensis*	*Coxiella*	2.6
*O. sawaii*	*Coxiella*	7.2
*O. moubata*	*Francisella*	64.2
	*Coxiella*	34.9

**Table 5:** Prevalence of bacterial taxa that include potential pathogens in the examined argasid ticks.

Bacteria	*A. japonicus*	*C. vespertilionis*	*O. capensis*	*O. sawaii*	*O. moubata*
*Ehrlichia*	0	0	4 (8.2%)	1 (5.0%)	0
*Borrelia*	0	0	3 (6.1%)	0	0
*Bartonella*	0	0	1 (2.0%)	0	0
*Diplorickettsiaceae*	0	1 (7.1%)	9 (18.4%)	1 (5.0%)	0

Although the microbiome of *O. capensis* was mainly dominated by commensal bacteria (Fig. S12), we found that the mean relative abundance of the potential endosymbiont *Coxiella* sp. was 2.6% and detected in 93.9% (46/49) of the examined *O. capensis* samples (Table 4). Similarly, commensals dominated the microbiome of *O. sawaii* samples (Fig. S13) and the mean relative abundance of *Coxiella* sp. was 7.2% detected from 100% (20/20) of the examined *O. sawaii* samples (Table 4). In contrast, the microbiome of the laboratory colony of *O. moubata* was dominated by the bacterial genera *Francisella* and *Coxiella* (Table 4 and Fig. S14) (mean relative abundance = 64.2% and 34.9%, respectively).

Other than the endosymbionts and commensals, we detected ASVs belonging to *Anaplasmataceae*, *Spirochaetaceae*, *Rhizobiaceae*, and *Diplorickettsiaceae* (Fig. 3 and Table 5). Among these potential pathogenic bacteria, we detected *Ehrlichia* from *O. capensis* (*n* = 4) and *O. sawaii* (*n* = 1), and *Borrelia* and *Bartonella* from *O. capensis* (*n* = 3 and 1, respectively) (Fig. 3).

LEfSe analysis detected differences in the community composition between the examined argasid species for the same sex and stage (Fig. S15-17). Far fewer discriminant bacterial taxa were detected within each argasid species when compared according to the sex and life stage. Although the majority of these bacterial taxa were commensals, we detected significant differential abundances for endosymbionts and pathogens in relation to sex and stage (Fig. 3 and Fig. S18-19). Notably, the relative abundance of *Occidentia* sp. was higher in female than male *A. japonicus.* Female *O. capensis* showed higher abundance of *Coxiella* sp. than in males from the same species. Interestingly, the abundance of *Francisella* sp. in the nymphal stage was significantly higher than in both male and female *O. moubata*. In contrast, adult *O. moubata* had higher abundance of *Coxiella* than nymphs.

### 3.4 *Isolation and molecular characterization of Occidentia massiliensis from Argas japonicus*

**Fig. 4:**
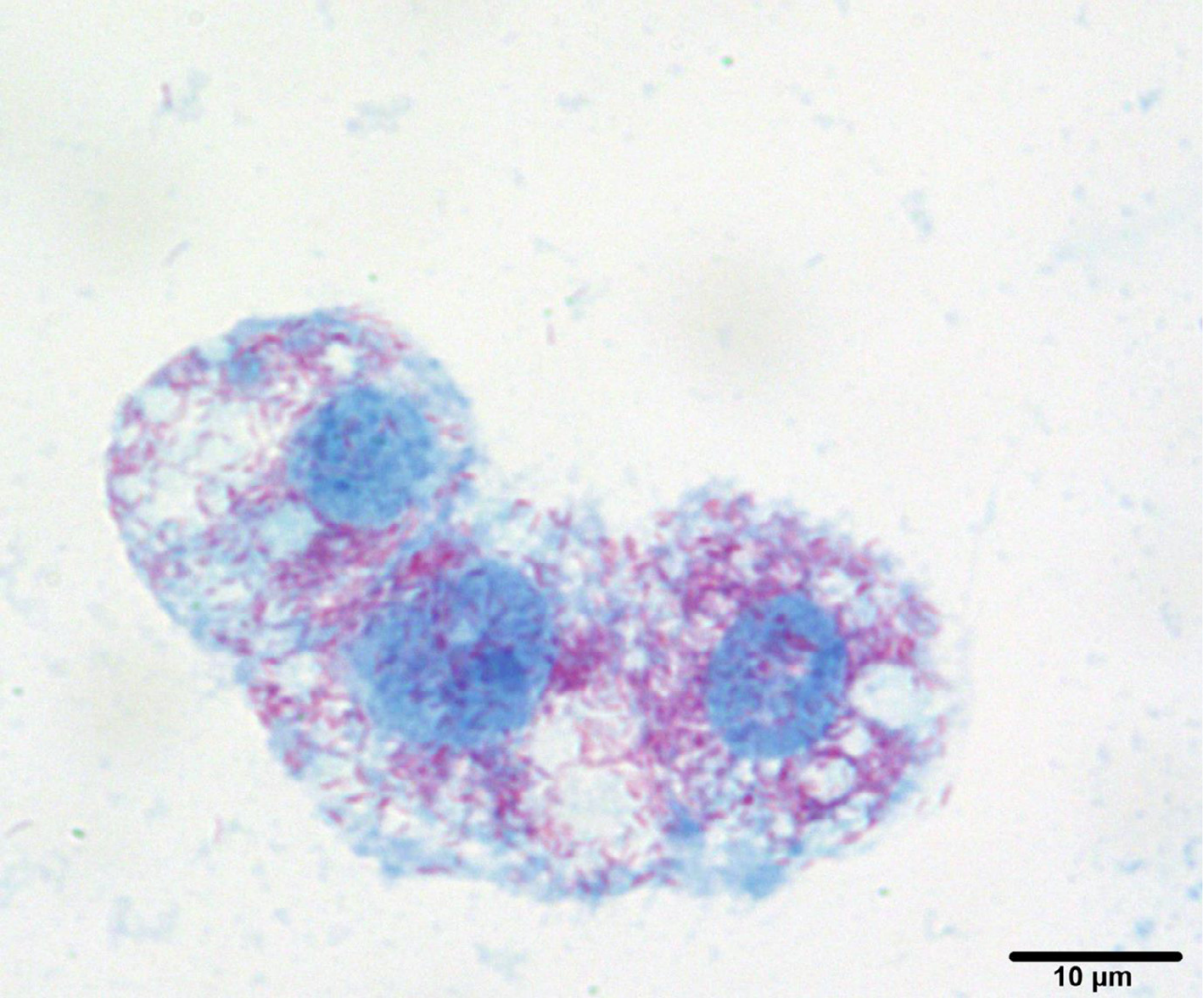
A Gimenez stained cytocentrifuged smear of *I. scapularis* cell line ISE6 infected with *Oc. massiliensis* isolated from *A. japonicus*.

**Fig. 5:**
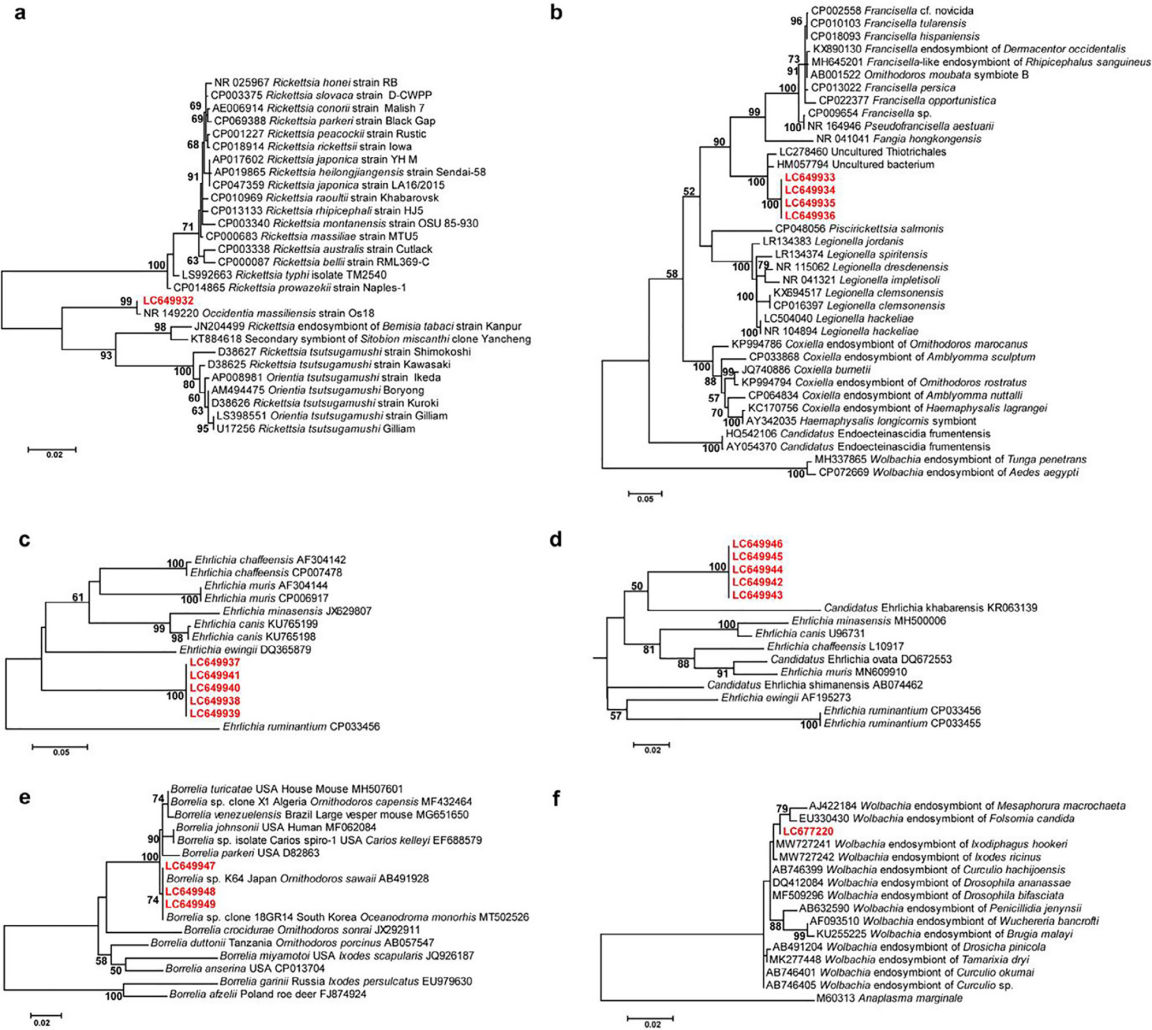
Molecular characterization of endosymbionts and pathogens in argasid ticks. The figure shows maximum likelihood phylogenetic analyses based on: (**a**) nearly full 16S rRNA gene sequence of *Occidentia* species, (**b**) nearly full 16S rRNA gene sequence of uncultured bacterium belonging to the order Thiotrichales, (**c**) partial *gltA* gene sequences of *Ehrlichia* species, (**d**) partial *groEL* gene sequences of *Ehrlichia* species, (**e**) partial *flaB* gene sequences of *Borrelia* species, and (**f**) nearly full 16S rRNA gene sequence of *Wolbachia* species. The branches were supported by 1,000 bootstrap replications.

During an 8-week incubation period, a bacterial isolate was obtained from one single *A. japonicus* sample inoculated into ISE6 cells (Fig. 4). A total of 14 and 27 samples were contaminated with environmental bacteria/fungi for ISE6 and C6/36 cells, respectively. The sequencing analysis of 16S rRNA gene PCR amplicon of the isolate (GenBank accession number: LC649932) showed 99% identity to *Oc. massiliensis* strain Os18 (GenBank accession number: NR_149220), which was isolated from *Ornithodoros sonari* from Senegal. The phylogenetic tree showed that the obtained sequence clustered with *Oc. massiliensis* and formed a separate clade between the previously published *Orientia* and *Rickettsia* species (Fig. 5a).

### 3.5 Molecular characterization of a potential novel endosymbiont belonging to the order Thiotrichales

Although this bacterium was identified as *Candidatus* Endoecteinascidia by the SILVA classifier in our taxonomy analysis, the obtained nearly full 16S rRNA gene sequences were identical to each other and showed 96% identity to uncultured Thiotrichales (GenBank accession number: LC278460) isolated from *Asterias amurensis* from Hokkaido, Japan. The phylogenetic tree showed that the obtained four sequences (GenBank accession numbers: LC649933-LC649936) clustered with the previously published sequences from uncultured bacteria belonging to the order Thiotrichales and formed a clade adjacent to the cluster of *Francisella* species (Fig. 5b).

### 3.6 Molecular characterization of potential novel pathogens in *Ornithodoros* species

The partial *gltA* gene sequences (GenBank accession numbers: LC649937-LC649941) of the detected *Ehrlichia* sp. (*n* = 5) were identical and showed 85.2% identity to *E. canis* (GenBank accession number: AY647155) reported from dogs in Italy. The partial *groEL* gene sequences (GenBank accession numbers: LC649942-LC649946) of the detected *Ehrlichia* sp. (*n* = 5) were identical and showed 91% identity to *Ehrlichia* sp. (GenBank accession number: KJ410295) from a tick (*Dermacentor nuttalli*) in China. The phylogenetic trees based on partial *gltA* and *groEL* gene sequences showed that the detected *Ehrlichia* sp. formed a new clade (Fig. 5c and Fig. 5d).

In addition, three *Borrelia* (from three *O. capensis*) and one *Wolbachia* (from *O. sawaii*) were detected in the examined samples. The obtained partial *flaB* gene sequences (GenBank accession numbers: LC649947-LC649949) of the detected *Borrelia* sp. were identical to each other and to *Borrelia* sp. K64 (GenBank accession number: AB491928) from *O. sawaii* in Japan and *Borrelia* sp. (GenBank accession number: MT502530) from *O. sawaii* in South Korea. The phylogenetic analysis showed that the detected *Borrelia* sp. sequences clustered together with *Borrelia turicatae* group (Fig. 5e). The obtained nearly full 16S rRNA gene sequence of *Wolbachia* sp. (GenBank accession number: LC677220) showed 99.4% identity to *Wolbachia* endosymbiont of *Folsomia candida* (GenBank accession number: EU330430) isolated from USA. The phylogenetic tree showed that the obtained sequence clustered with the previously published sequences from a *Wolbachia* endosymbiont of other arthropods (Fig. 5f).

## 4 Discussion

During the past decade, the research on vector-associated microbiomes has been largely expanding due to improvements of sequencing technology and bioinformatic tools in this field that has contributed greatly to better understanding of tick systematics [86], [87] and the emergence of novel TBPs [88]. However, little is known about microbiomes of argasid ticks and the drivers affecting its composition [41]. In our study, microbiomes of 137 argasid ticks of 5 different species were investigated and the association with species and sex/stage variations in Japan was explained. Two novel putative endosymbionts, together with novel *Ehrlichia*, *Borrelia*, and *Wolbachia* were molecularly characterized among these argasid samples.

Although our study did not include field collected *O. moubata* samples, the examined laboratory colony showed significantly lower number of features as compared to those in the field collected samples from the other four species. The difference in microbiome between field and laboratory collected ticks has previously been demonstrated in *Amblyomma maculatum* [89], where the microbiome diversity was higher in the field collected *A. maculatum* than in the laboratory reared ones. In another study, laboratory colonization of *Dermacentor andersoni* resulted in stabilization of the associated microbiome [90]. Hence, our results support the hypothesis that laboratory colonization of ticks can reduce the role of ecological factors in changing the associated microbiome in a way that can improve the biological control of ticks by targeting the essential remaining endosymbionts after colonization. This strategy has helped in the production of germ-free mosquitoes and the acceleration of the study of microbiome in insects [91].

Few studies have demonstrated the effect of species variation on argasid tick microbiome [10], [22], [92] due to diverse animal hosts and geographical distribution. We have showed that the differences in microbiome composition and richness in this study were best explained by variations in tick species. This strong species-specific variation in the microbiome of argasid ticks can be attributed to differences in collection sites [93], [94] as well as potential animal hosts [95]. In this study, samples representing each argasid species were collected from a specific collection site, which hindered testing the effect of spatial variation within the same species. Recorded host preferences are variable among the argasid species employed in this study. Briefly, the host animals for *A. japonicus*, *C. vespertilionis*, *O. capensis*, and *O. sawaii* are the common house martin (*Delichon urbicum*), the Japanese short-tailed bat (*Ep. japonensis*), the wedge-tailed shearwater (*Puffinus pacificus*), and the Swinhoe's petrel (*Oce. monorhis*) [96], [97], respectively. This animal host variation may explain the significant differences in microbiome composition and richness between the examined species of ticks. In general, argasid ticks feed several times from their animal hosts during their life cycles [98]. Hence, many commensal bacteria and pathogens can be transmitted to these ticks from the skin and blood of their animal hosts during feeding [13], [14]. The taxonomy composition analysis showed that *Coxiella* was the potential endosymbiont for 4 out of 5 argasid species. Only *A. japonicus* was dominated either by *Oc. massiliensis* (11/38) or an uncultured Thiotrichales bacterium (27/38). In addition, dual symbiosis was observed between *Rickettsia* and *Coxiella* or *Francisella* and *Coxiella* in *C. vespertilionis* and *O. moubata*, respectively. Some of the members of *Coxiella*, *Rickettsia*, and *Francisella* (obligate intracellular bacteria transmitted vertically) have a major nutritional role in ticks and have been detected from ixodid and argasid ticks [13], [47], [99], [100], [101], [102]. Previously, high dual abundance of *Rickettsia* and *Coxiella* was detected in *O. capensis* and *Ornithodoros maritimus* [41], [48]. Furthermore, experimental elimination of *Francisella* endosymbiont in *O. moubata* has prevented the development of adult females [101].

Differences in microbiome were not only limited to between different argasid species but was also detected among individual ticks belonging to the same species (Fig. 2 and Fig. S10-14). These differences may be due to extrinsic or intrinsic variables such as the tick general physiological health, blood meal sources and histories, presence or absence of pathogenic bacteria and protozoa, tick life stage and sex, and tick genetic backgrounds [26], [103], [104], [105]. Our study design cannot illustrate all these factors to explain this variability among individuals from the same species, but we showed that infection with potential pathogens, sex, and stage can contribute to this pattern of microbiome variability.

Generally, co-occurrence of endosymbionts in one host may have several potential beneficiary consequences such as nutritional and protective functions [106]. For example, *Midichloria* could compensate the biotin biosynthesis pathways that are deficient in the co-symbiont *Francisella* in *Hyalomma marginatum* [107]. Another example is the agonist interaction between two endosymbionts through synthesizing different important nutrients in the glassy-winged sharpshooter (*Homalodisca coagulata*) [108]. In *Aphidius ervi*, the primary symbiont, *Buchnera aphidicola*, contributes mainly to the nutritional needs of the host, whereas the secondary symbiont, *'Candidatus* Hamiltonella defensa', encodes toxins that protect the aphid from parasitoids [109]. In ixodid ticks, *Rickettsia*, *Coxiella*, and *Francisella* endosymbionts were found to co-exist in *Dermacentor reticulatus* and *Ixodes ricinus* collected in Slovakia [110]. Furthermore, a comparison between *Coxiella* and *Francisella* endosymbionts from ixodid ticks has revealed that their metabolism is highly similar, but only *Francisella* can produce more products than *Coxiella* including cysteine, threonine, tyrosine, tryptophan, phenylalanine, serine, asparagine, glutamine, proline, and heme, while *Coxiella* generates thiamine [111]. We assume that bacterial endosymbionts in argasid ticks are highly related to the blood meal source, especially that the metabolites provided are highly variable between the nucleated red blood cells (RBCs) in birds and reptiles, and the non-nucleated RBCs in mammals [112]. This can be supported by our results that *C. vespertilionis* and *O. moubata* ticks, which feed on blood meals containing mostly non-nucleated cells from Japanese short-tailed bats and mammals, were associated with dual symbiosis while the remaining three argasid ticks, which feed on blood meals containing mostly nucleated cells from birds, were associated with a single potential bacterial symbiont. Future investigations are required to confirm the effect of the blood meal source on the evolution of bacterial symbiosis in argasid ticks.

Previously, *Oc. massiliensis* was isolated from *O. sonrai* collected from rodent burrows in Senegal [113] and from *Africaniella transversale* collected from *Python regius* imported from Senegal to the United Arab Emirates [114], but no clear information was provided on its role as an endosymbiont. Because *O. sonrai* and *Af. transversale* are known to infest reptilian hosts during their life cycles [115], [116], it appears that pythons and rodents (pythons’ main prey) are equally susceptible to *Oc. massiliensis* infections. However, our study has isolated and characterized *Oc. massiliensis* for the first time from *A. japonicus* samples collected from Japan, suggesting a wider geographical distribution and host range of this endosymbiont than previously expected. We showed that 16S rRNA gene sequences from the isolated *Oc. massiliensis* is closely related to *Rickettsia* endosymbionts of the silverleaf whitefly (*Bemisia tabaci*) [117] and the Indian grain aphid (*Sitobion miscanthi*) [118], [119], which supports the possibility that this bacterium can have a role as an endosymbiont in *A. japonicus*. Although we were not successful in isolating the other potential endosymbionts of *A. japonicus* that belong to the order Thiotrichales, we showed that this uncultured bacterium 16S rRNA gene sequence is closely related to the clade containing *Francisella* spp. that are considered as endosymbionts of several *Argas* species [120].

A previous study showed that the microbiome of female *Ixodes* ticks was less diverse than in males [94] due to the high abundance of *Rickettsia* species in females [80]. Although we have not included larvae in our investigation, we detected significant correlations between the life stage and the microbiome diversity in *O. capensis* and *O. moubata*. The explanation of this correlation may depend on the balance between the acquired environmental bacteria and the burden of endosymbionts within argasid ticks. This can explain the inconsistent alpha diversity values between different life stages in the tick species of this study (Fig. 1 and S6). We showed that the nymphal stage of *O. capensis* and *O. moubata* presented lower alpha diversity values than adults, but no significant correlations were detected within the remaining argasids according to sex and stage variations. There are contrasting opinions regarding the interpretation of the correlation between life stage and microbiome diversity in ticks. One opinion is that there are positive correlations between alpha diversity and the tick life stage [121], except that females have lower diversity due to the colonization by endosymbionts such as *Rickettsia* species that lead to competition with other bacteria for resources [22], [122]. Another opinion is that alpha diversity decreases by tick life stage due to the dilution effect through bloodmeal feeding in *I. pacificus* [123]. Our results from four field collected and one laboratory colony of argasid ticks suggest that it is difficult to find a consistent trend of microbiome diversity in relation to tick life stage that fits all tick species, and the final interpretation should be related to the life history of the samples and the balance between environmental bacterial exposure and endosymbiont ecology.

The LEfSe analysis showed that adult females of *O. capensis* and *O. moubata* have significantly higher abundance of *Coxiella*. Interestingly, the relative abundance of *Francisella* in *O. moubata* was significantly higher in nymphs than in adults. The relationship between *Francisella* and *Coxiella* in *O. moubata* with the life stage is not fully covered but it was suggested that *Francisella* has replaced *Coxiella* in some ticks [44], [124]. A similar trend was observed in *Amblyomma americanum* where males harbored higher abundance of *Rickettsia* than females wihch had higher levels of *Coxiella* endosymbionts [125]. This may indicate that males have higher numbers of *Rickettsia* to facilitate locomotion to increase the mating opportunities [126]. However, it is not clear yet if *Francisella* has any role in locomotion facilitation in argasids. Although we detected significant variations in microbial diversity and richness among several developmental stages and sexes within the same argasid tick species, our analysis revealed that some bacterial dynamics are solid throughout the tick life stage. Indeed, argasid ticks are nidicolous but can disperse among several colonies of seabird species during the movement of their hosts [127], where new bacterial species can be acquired from the different environment [41].

Most of the previously published studies on *Ehrlichia* species have focused on ixodid ticks and animals [128], [129]. We detected *Ehrlichia* from *O. capensis* and *O. sawaii* which showed 85 – 91% identity to the previously published *gltA* and *groEL* gene sequences of *Ehrlichia* species. The number of the reports on uncultured *Ehrlichia* spp. has been increasing worldwide. Recently, a study has characterized a total of 11 newly identified *Ehrlichia* genotypes detected in *Haemaphysalis* ticks in Japan [130]. To the best of our knowledge, this is the first study to report *Ehrlichia* spp. from argasid ticks in Japan. A closely related species to *Candidatus* Ehrlichia khabarensis has been detected from *C. vespertilionis* in the UK [36] and a new species related to *E. canis* group (*Ehrlichia* sp. AvBat) was detected from the same tick species from France [131]. Due to the relative lack of available data regarding the prevalence of *Ehrlichia* spp. in *Ornithodoros* ticks, more investigations are needed in the future to identify the role of argasid ticks as vectors for TBPs and the pathogenicity of the detected *Ehrlichia* sp. to humans and animals.

Several bacterial TBDs, such as Japanese spotted fever, Lyme disease, relapsing fever, and scrub typhus, are endemic in Japan [132]. We detected a *Borrelia* sp. in *O. capensis* that clustered with the relapsing fever *Borrelia* group including *Borrelia parkeri*, *B. turicatae*, *Borrelia venezuelensis*, and *Borrelia johnsonii.* Previous studies in Japan have detected several Lyme disease borreliae (eg, *Borrelia japonica*) and relapsing fever borreliae (eg, *Borrelia miyamotoi*) in ixodid ticks [133], [134], [82]. In addition, *Borrelia* sp. K64 has an identical partial *flaB* gene sequence with the one obtained in our study, and it was reported from *O. sawaii* in Japan [135]. This *Borrelia* sp. has been detected recently from *O. sawaii* in South Korea [97], but our study is the first to detect this species in *O. capensis*. These findings support the possibility that migratory seabirds are the vertebrate hosts for this *Borrelia* species. Additionally, the presence of *Borrelia* sp. closely related to *B. turicatae* was highlighted in *O. capensis* from Algeria, which suggests that this *Borrelia* sp. is maintained by seabirds and transmitted by the associated argasid ticks [136]. Hence, more investigations are required to know if this *Borrelia* sp. could represent a public health threat in Japan and South Korea.

*Wolbachia* species are highly diverse in invertebrates and represent an important group of endosymbionts in nematodes and insects [137], [138], [139]. Our study is the first to detect a *Wolbachia* sp. in *O. sawaii*. Previously, several *Wolbachia* spp. were detected from *I. ricinus* from France, Germany, Italy, and The Netherlands [140] and it was suggested that this bacterium could have negative impact on the tick microbiome due to the induction of immune reactions [141]. The presence of *Wolbachia* sp. in one of our *O. sawaii* samples can be due to the association of this argasid sample with an endoparasitoid as was shown for *I. ricinus* samples infected with *Wolbachia* sp. [142] due to the infestation by *Ixodiphagus hookeri* (Hymenoptera, Chalcidoidea, Encyrtidae). Although we did not investigate the presence of endoparasitoids in our samples, the phylogenetic analysis showed that the detected *Wolbachia* sp. in our study is closely related to the endosymbiont of *I. hookeri*, which supports the hypothesis that this argasid tick sample was probably infested with an endoparasitoid species that uses this *Wolbachia* sp. as an endosymbiont.

## 5 Conclusions

By targeting the bacterial communities in 137 argasid ticks collected from multiple locations in Japan, we were able to provide insights on the microbiome of four natural and one laboratory reared argasid species. We showed that the microbial community structures within each argasid species is distinct from the other and can be affected by life stage variation. Furthermore, our study illustrated that only a few bacterial genera differ in abundance within the same argasid species for different life stages. Specifically, *Occidentia*, *Coxiella*, and *Francisella* endosymbionts showed significant correlations with the argasid tick life stage variations. We documented the isolation and molecular characterization of novel endosymbionts in *A. japonicus*, including *Oc. massiliensis* and a bacterium belonging to the order Thiotrichales. The two newly identified potential symbionts expand the current identified phylogenetic clades of tick endosymbionts. We detected and characterized a potentially novel *Ehrlichia* sp. from *O. capensis* and *O. sawaii*. Additionally, we detected a relapsing fever *Borrelia* sp. for the first time from *O. capensis*. Our study should encourage future investigations on argasid ticks to enrich our understanding about microbiome ecology and possible manipulations to provide next generation control strategies for ticks. Likewise, investigations are needed to identify the reservoir capacity of argasid ticks to TBPs with zoonotic and veterinary importance, considering proper isolation and characterization of the detected pathogens.

## 6 Availability of data and materials

The raw sequence data were deposited in the DNA Data Bank of the Japan Sequence Read Archive under the DRA accession number: DRA013238.

## Funding

This research was supported by KAKENHI (16H06431, 19H03118, 19F19097, 20K21358 and 20KK0151) and the Japan Program for Infectious Diseases Research and Infrastructure (21wm0225016j0002) from the Japan Agency for Medical Research and Development (AMED).

## CRediT authorship contribution statement

**Mohamed Abdallah Mohamed Moustafa:** Investigations, methodology and writing the main manuscript. **Wessam Mohamed Ahmed Mohamed:** Bioinformatics. **Alice C.C. Lau:** Methodology. **Elisha Chatanga:** Methodology. **Yongjin Qiu:** Methodology. **Naoki Hayashi:** Methodology. **Doaa Naguib:** Methodology. **Kozue Sato:** Methodology. **Ai Takano:** Methodology. **Keita Mastuno:** Methodology. **Nariaki Nonaka:** Provided the materials and resources. **DeMar Taylor:** Provided the materials and resources. **Hiroki Kawabata:** Provided the materials and resources. **Ryo Nakao:** Supervision andwriting the main manuscript.

## Declaration of Competing Interest

The authors declare that they have no known competing financial interests or personal relationships that could have appeared to influence the work reported in this paper.

## References

[1] Socolovschi C., Mediannikov O., Raoult D., Parola P. (2009). The relationship between spotted fever group Rickettsiae and ixodid ticks. Vet Res.

[2] Villar M., Pacheco I., Merino O., Contreras M., Mateos-Hernandez L., Prado E. (2020). Tick and host derived compounds detected in the cement complex substance. Biomolecules.

[3] Van Nunen S.A., O'Connor K.S., Clarke L.R., Boyle R.X., Fernando S.L. (2009). An association between tick bite reactions and red meat allergy in humans. Med J Aust.

[4] Hall-Mendelin S., Craig S.B., Hall R.A., O'Donoghue P., Atwell R.B., Tulsiani S.M. (2011). Tick paralysis in Australia caused by *Ixodes holocyclus* Neumann. Ann Trop Med Parasitol.

[5] Parola P., Raoult D. (2001). Ticks and tickborne bacterial diseases in humans: an emerging infectious threat. Clin Infect Dis.

[6] Chapter 4 - Arachnida, in: Marchiondo A.A., Cruthers L.R., Fourie J.J., editor. Parasiticide screening (Volume 1). Academic Press; 2019. p. 257-377.

[7] Sonenshine D.E., Roe R.M. (2013).

[8] Dantas-Torres F., Chomel B.B., Otranto D. (2012). Ticks and tick-borne diseases: a One Health perspective. Trends Parasitol.

[9] Nava S., Guglielmone A.A., Mangold A.J. (2009). An overview of systematics and evolution of ticks. Front Biosci (Landmark edition).

[10] Karim S., Budachetri K., Mukherjee N., Williams J., Kausar A., Hassan M.J. (2017). A study of ticks and tick-borne livestock pathogens in Pakistan. Plos Neglect Trop Dis.

[11] Ben-Yosef M., Rot A., Mahagna M., Kapri E., Behar A., Gottlieb Y. (2020). *Coxiella*-Like endosymbiont of *Rhipicephalus sanguineus* is required for physiological processes during ontogeny. Front Microbiol.

[12] Moran N.A., McCutcheon J.P., Nakabachi A. (2008). Genomics and evolution of heritable bacterial symbionts. Annu Rev Genet.

[13] Bonnet S.I., Pollet T. (2021). Update on the intricate tango between tick microbiomes and tick-borne pathogens. Parasite Immunol.

[14] Narasimhan S., Fikrig E. (2015). Tick microbiome: the force within. Trends Parasitol.

[15] Khoo J.-J., Chen F., Kho K.L., Ahmad Shanizza A.I., Lim F.-S., Tan K.-K. (2016). Bacterial community in *Haemaphysalis* ticks of domesticated animals from the Orang Asli communities in Malaysia. Ticks Tick-Borne Dis.

[16] Telfer S., Lambin X., Birtles R., Beldomenico P., Burthe S., Paterson S. (2010). Species interactions in a parasite community drive infection risk in a wildlife population. Science.

[17] Yan P., Qiu Z., Zhang T., Li Y., Wang W., Li M. (2019). Microbial diversity in the tick *Argas japonicus* (Acari: Argasidae) with a focus on *Rickettsia* pathogens. Med Vet Entomol.

[18] Rojas-Jaimes J., Lindo-Seminario D., Correa-Núñez G., Diringer B. (2021). Characterization of the bacterial microbiome of *Rhipicephalus (Boophilu*s) *microplu*s collected from *Pecari tajacu* “Sajino” Madre de Dios, Peru. Sci Rep.

[19] Thapa S., Zhang Y., Allen M.S. (2019). Bacterial microbiomes of *Ixodes scapularis* ticks collected from Massachusetts and Texas, USA. BMC Microbiol.

[20] de la Fuente J., Antunes S., Bonnet S., Cabezas-Cruz A., Domingos A.G., Estrada-Peña A., et al. Tick-pathogen interactions and vector competence: identification of molecular drivers for tick-borne diseases. Front Cell Infect Microbiol 2017;7:114–114. 10.3389/fcimb.2017.00114 PMC5383669 28439499

[21] Aivelo T., Norberg A., Tschirren B. (2019). Bacterial microbiota composition of *Ixodes ricinus* ticks: the role of environmental variation, tick characteristics and microbial interactions. PeerJ.

[22] Kueneman J.G., Esser H.J., Weiss S.J., Jansen P.A., Foley J.E. (2021). Tick microbiomes in neotropical forest fragments are best explained by tick-associated and environmental factors rather than host blood source. Appl Environ Microbiol.

[23] Wu-Chuang A., Obregon D., Estrada-Peña A., Cabezas-Cruz A. (2021). Thermostable keystone bacteria maintain the functional diversity of the *Ixodes scapularis* microbiome under heat stress. Microb Ecol.

[24] Estrada-Peña A., Cabezas-Cruz A., Obregón D. (2020). Resistance of tick gut microbiome to anti-tick vaccines, pathogen infection and antimicrobial peptides. Pathogens.

[25] Brinkerhoff R.J., Clark C., Ocasio K., Gauthier D.T., HynesW.L. Factors affecting the microbiome of *Ixodes scapularis* and *Amblyomma americanum*. PLoS One 2020;15(5):e0232398-e0232398. 10.1371/journal.pone.0232398 PMC7228056 32413031

[26] Bonnet S.I., Binetruy F., Hernández-Jarguín A.M., Duron O. The Tick Microbiome: Why non-pathogenic microorganisms matter in tick biology and pathogen transmission. Front Cell Infect Microbiol 2017;7:236–236. 10.3389/fcimb.2017.00236 PMC5462901 28642842

[27] Mans B.J., Kelava S., Pienaar R., Featherston J., de Castro M.H., Quetglas J. (2021). Nuclear (18S–28S rRNA) and mitochondrial genome markers of *Carios (Cario*s) *vespertilioni*s (Argasidae) support Carios Latreille, 1796 as a lineage embedded in the Ornithodorinae: re-classification of the *Cario*s sensu Klompen and Oliver (1993) clade into its respective subgenera. Ticks Tick-Borne Dis.

[28] Munoz-Leal S., Terassini F.A., Marcili A., Oliveira G.M.B., Camargo L.M.A., Labruna M.B. A third species of Nothoaspis Keirans & Clifford, 1975 (Acari: Argasidae): Nothoaspis setosus (Kohls, Clifford & Jones, 1969) n. comb. Syst Parasitol 2019;96(7):595-602. 10.1007/s11230-019-09873-9 31367960

[29] Munoz-Leal S., Venzal J.M., Nava S., Marcili A., Gonzalez-Acuna D., Martins T.F. (2020). Description of a new soft tick species (Acari: Argasidae: *Ornithodoros*) parasite of *Octodon degus* (Rodentia: Octodontidae) in northern Chile. Ticks Tick-Borne Dis.

[30] Ehlers J., Kruger A., Rakotondranary S.J., Ratovonamana R.Y., Poppert S., Ganzhorn J.U. (2020). Molecular detection of *Rickettsia* spp., *Borrelia* spp., *Bartonella* spp. and *Yersinia* pestis in ectoparasites of endemic and domestic animals in southwest Madagascar. Acta Trop.

[31] Zhao S., Yang M., Liu G., Hornok S., Zhao S., Sang C. (2020). Rickettsiae in the common pipistrelle *Pipistrellus pipistrellus* (Chiroptera: Vespertilionidae) and the bat soft tick *Argas vespertilionis* (Ixodida: Argasidae). Parasites Vectors.

[32] Qiu Y., Simuunza M., Kajihara M., Chambaro H., Harima H., Eto Y. (2021). Screening of tick-borne pathogens in argasid ticks in Zambia: Expansion of the geographic distribution of *Rickettsia lusitaniae* and *Rickettsia hoogstraalii* and detection of putative novel *Anaplasma* species. Ticks Tick-Borne Dis.

[33] Ouchene N., Nebbak A., Ouchene-Khelifi N.A., Dahmani A., Zeroual F., Khelef D. (2020). Molecular detection of avian spirochete *Borrelia anserina* in *Argas persicus* ticks in Algeria. Comp Immunol Microbiol Infect Dis.

[34] Sambri V., Marangoni A., Storni E., Cavrini F., Moroni A., Sparacino M. (2004). Tick borne zoonosis: selected clinical and diagnostic aspects. Parassitologia.

[35] Qiu Y., Nakao R., Hang'ombe B.M., Sato K., Kajihara M., Kanchela S. (2019). Human borreliosis caused by a new world relapsing fever *Borrelia*-like organism in the old world. Clin Infect Dis.

[36] Lv J., Fernandez de Marco M.D.M., Goharriz H., Phipps L.P., McElhinney L.M., Hernandez-Triana L.M. (2018). Detection of tick-borne bacteria and babesia with zoonotic potential in *Argas (Cario*s) *vespertilioni*s (Latreille, 1802) ticks from British bats. Sci Rep.

[37] Jia N., Wang J., Shi W., Du L., Sun Y., Zhan W. (2020). Large-scale comparative analyses of tick genomes elucidate their genetic diversity and vector capacities. Cell.

[38] Herms W., Wheeler C. The tick vector of the infection, Relapsing fever. California State Department of Public Health Special Bull 1936;1936:24–28.

[39] Mans B.J., Featherston J., Kvas M., Pillay K.-A., de Klerk D.G., Pienaar R. (2019). Argasid and ixodid systematics: Implications for soft tick evolution and systematics, with a new argasid species list. Ticks Tick-Borne Dis.

[40] Dupraz M., Toty C., Devillers E., Blanchon T., Elguero E., Vittecoq M. (2017). Population structure of the soft tick *Ornithodoros maritimus* and its associated infectious agents within a colony of its seabird host *Larus michahellis*. Int J Parasitol Parasites Wildl.

[41] Gomard Y., Flores O., Vittecoq M., Blanchon T., Toty C., Duron O. (2021). Changes in bacterial diversity, composition and interactions during the development of the seabird tick *Ornithodoros maritimus* (Argasidae). Microb Ecol.

[42] Kwak M.L. (2018). A checklist of the ticks (Acari: Argasidae, Ixodidae) of Japan. Exp Appl Acarol.

[43] Ohashi N., Gaowa W., Kawamori F., Wu D., Yoshikawa Y., Chiya S. (2013). Emerg Infect Dis.

[44] Duron O., Binetruy F., Noel V., Cremaschi J., McCoy K.D., Arnathau C. (2017). Evolutionary changes in symbiont community structure in ticks. Mol Ecol.

[45] Ponnusamy L., Gonzalez A., Van Treuren W., Weiss S., Parobek C.M., Juliano J.J. (2014). Diversity of Rickettsiales in the microbiome of the lone star tick, *Amblyomma americanum*. Appl Environ Microbiol.

[46] Gofton A.W., Oskam C.L., Lo N., Beninati T., Wei H., McCarl V. (2015). Inhibition of the endosymbiont “*Candidatus* Midichloria mitochondrii” during 16S rRNA gene profiling reveals potential pathogens in *Ixodes* ticks from Australia. Parasites Vectors.

[47] Barraza-Guerrero S.I., Meza-Herrera C.A., Garcia-De la Pena C., Gonzalez-Alvarez V.H., Vaca-Paniagua F., Diaz-Velasquez C.E. (2020). General microbiota of the soft tick *Ornithodoros turicata* parasitizing the Bolson tortoise (*Gopherus flavomarginatus*) in the Mapimi biosphere reserve, Mexico. Biology (Basel).

[48] Wilkinson D.A., Dietrich M., Lebarbenchon C., Jaeger A., Le Rouzic C., Bastien M. (2014). Massive infection of seabird ticks with bacterial species related to *Coxiella burnetii*. Appl Environ Microbiol.

[49] Noda H., Munderloh U.G., Kurtti T.J. (1997). Endosymbionts of ticks and their relationship to *Wolbachia* spp. and tick-borne pathogens of humans and animals. Appl Environ Microbiol.

[50] Burgdorfer W., Owen C.R. (1956). Experimental studies on argasid ticks as possible vectors of tularemia. J Infect Dis.

[51] Hoogstraal H., Clifford C.M., Keirans J.E., Kaiser M.N., Evans D.E. (1976). The *Ornithodoros* (*Alectorobius*) *capensis* group (Acarina: Ixodoidea: Argasidae) of the palearctic and oriental regions. *O. (A*.) *maritimu*s: identity, marine bird hosts, virus infections, and distribution in western Europe and northwestern Africa. J Parasitol.

[52] Uspensky I. (2005).

[53] Thu M.J., Qiu Y., Kataoka-Nakamura C., Sugimoto C., Katakura K., Isoda N. (2019). Isolation of *Rickettsia*, *Rickettsiella*, and *Spiroplasma* from questing ticks in Japan using arthropod cells. Vector-Borne Zoonotic Dis.

[54] Klindworth A., Pruesse E., Schweer T., Peplies J., Quast C., Horn M. (2013). Evaluation of general 16S ribosomal RNA gene PCR primers for classical and next-generation sequencing-based diversity studies. Nucleic Acids Res.

[55] Herlemann D.P., Labrenz M., Jürgens K., Bertilsson S., Waniek J.J., Andersson A.F. (2011). Transitions in bacterial communities along the 2000 km salinity gradient of the Baltic Sea. ISME J.

[56] Bolyen E., Rideout J.R., Dillon M.R., Bokulich N.A., Abnet C.C., Al-Ghalith G.A. (2019). Reproducible, interactive, scalable and extensible microbiome data science using QIIME 2. Nat Biotechnol.

[57] Callahan B.J., McMurdie P.J., Rosen M.J., Han A.W., Johnson A.J., Holmes S.P. (2016). DADA2: High-resolution sample inference from Illumina amplicon data. Nat Methods.

[58] Katoh K., Standley D.M. (2013). MAFFT multiple sequence alignment software version 7: improvements in performance and usability. Mol Biol Evol.

[59] Price M.N., Dehal P.S., Arkin A.P. (2010). FastTree 2–approximately maximum-likelihood trees for large alignments. PLoS One.

[60] Shannon C.E. (1948). A mathematical theory of communication. Bell Syst Tech J.

[61] Faith D.P. (1992). Conservation evaluation and phylogenetic diversity. Biol Conserv.

[62] DeSantis T.Z., Hugenholtz P., Larsen N., Rojas M., Brodie E.L., Keller K. (2006). Greengenes, a chimera-checked 16S rRNA gene database and workbench compatible with ARB. Appl Environ Microbiol.

[63] Pielou E.C. (1966). The measurement of diversity in different types of biological collections. J Theor Biol.

[64] McMurdie P.J., Holmes S. (2013). phyloseq: an R package for reproducible interactive analysis and graphics of microbiome census data. PLoS One.

[65] Lozupone C., Knight R. (2005). UniFrac: a new phylogenetic method for comparing microbial communities. Appl Environ Microbiol.

[66] Lozupone C.A., Hamady M., Kelley S.T., Knight R. (2007). Quantitative and qualitative beta diversity measures lead to different insights into factors that structure microbial communities. Appl Environ Microbiol.

[67] Jaccard P. (1908). Nouvelles recherches sur la distribution florale. Bull Soc Vaud Sci Nat.

[68] Sorensen T.A. (1948). A method of establishing groups of equal amplitude in plant sociology based on similarity of species content and its application to analyses of the vegetation on Danish commons. Biol Skar.

[69] Vazquez-Baeza Y., Pirrung M., Gonzalez A., Knight R. (2013). EMPeror: a tool for visualizing high-throughput microbial community data. GigaScience.

[70] Bokulich N.A., Kaehler B.D., Rideout J.R., Dillon M., Bolyen E., Knight R. (2018). Optimizing taxonomic classification of marker-gene amplicon sequences with QIIME 2’s q2-feature-classifier plugin. Microbiome.

[71] Davis N.M., Proctor D.M., Holmes S.P., Relman D.A., Callahan B.J. (2018). Simple statistical identification and removal of contaminant sequences in marker-gene and metagenomics data. Microbiome.

[72] Team R.C (2020). R A language and environment for statistical computing, R Foundation for Statistical. Computing.

[73] Hunter J.D. (2007). Matplotlib: A 2D graphics environment. Comput Sci Eng.

[74] Segata N., Izard J., Waldron L., Gevers D., Miropolsky L., Garrett W.S. (2011). Metagenomic biomarker discovery and explanation. Genome Biol.

[75] Houwenhuyse S., Stoks R., Mukherjee S., Decaestecker E. (2021). Locally adapted gut microbiomes mediate host stress tolerance. ISME J.

[76] Lüdecke D., Ben-Shachar M., Patil I., Waggoner P.M.D. (2021). Performance: An R package for assessment, comparison and testing of statistical models. J Open Source Software.

[77] Searle S.R., Speed F.M., Milliken G.A. (1980). Population marginal means in the linear model: An alternative to least squares means. Am Stat.

[78] Anderson M.J. (2001). A new method for non-parametric multivariate analysis of variance. Austral Ecol.

[79] Weisburg W.G., Barns S.M., Pelletier D.A., Lane D.J. (1991). 16S ribosomal DNA amplification for phylogenetic study. J Bacteriol.

[80] Qiu Y., Kaneko C., Kajihara M., Ngonda S., Simulundu E., Muleya W. (2018). Tick-borne haemoparasites and *Anaplasmataceae* in domestic dogs in Zambia. Ticks Tick-Borne Dis.

[81] Takano A., Goka K., Une Y., Shimada Y., Fujita H., Shiino T. (2010). Isolation and characterization of a novel *Borrelia* group of tick-borne borreliae from imported reptiles and their associated ticks. Environ Microbiol.

[82] Lee K., Takano A., Taylor K., Sashika M., Shimozuru M., Konnai S. (2014). A relapsing fever group *Borrelia* sp. similar to *Borrelia lonestari* found among wild sika deer (*Cervus nippon yesoensis*) and *Haemaphysalis* spp. ticks in Hokkaido, Japan. Ticks Tick-Borne Dis.

[83] Brown G., Martin A., Roberts T., Aitken R. (2001). Detection of *Ehrlichia platys* in dogs in Australia. Aust Vet J.

[84] Kumar S., Stecher G., Li M., Knyaz C., Tamura K. (2018). MEGA X: Molecular evolutionary genetics analysis across computing platforms. Mol Biol Evol.

[85] Guindon S., Dufayard J.F., Lefort V., Anisimova M., Hordijk W., Gascuel O. (2010). New algorithms and methods to estimate maximum-likelihood phylogenies: assessing the performance of PhyML 3.0. Syst Biol.

[86] Kurokawa C., Lynn G.E., Pedra J.H.F., Pal U., Narasimhan S., Fikrig E. (2020). Interactions between *Borrelia burgdorferi* and ticks. Nat Rev Microbiol.

[87] Wu-Chuang A., Hodžić A., Mateos-Hernández L., Estrada-Peña A., Obregon D., Cabezas-Cruz A. (2021). Current debates and advances in tick microbiome research. Curr Res Parasitol Vector-Borne Dis.

[88] Merenstein C., Ward J., Allen D. (2020). *Diplorickettsia* bacteria in an *Ixodes scapularis* tick, Vermont, USA. Emerg Infect Dis.

[89] Budachetri K., Browning R.E., Adamson S.W., Dowd S.E., Chao C.C., Ching W.M. (2014). An insight into the microbiome of the *Amblyomma maculatum* (Acari: Ixodidae). J Med Entomol.

[90] Gall C.A., Scoles G.A., Magori K., Mason K.L., Brayton K.A. (2017). Laboratory colonization stabilizes the naturally dynamic microbiome composition of field collected *Dermacentor andersoni* ticks. Microbiome.

[91] Romoli O., Schönbeck J.C., Hapfelmeier S., Gendrin M. (2021). Production of germ-free mosquitoes via transient colonisation allows stage-specific investigation of host–microbiota interactions. Nat Commun.

[92] Chicana B., Couper L.I., Kwan J.Y., Tahiraj E., Swei A. (2019). Comparative microbiome profiles of sympatric tick species from the far-western United States. Insects.

[93] Pollet T., Sprong H., Lejal E., Krawczyk A.I., Moutailler S., Cosson J.-F. (2020). The scale affects our view on the identification and distribution of microbial communities in ticks. Parasites Vectors.

[94] Van Treuren W., Ponnusamy L., Brinkerhoff R.J., Gonzalez A., Parobek C.M., Juliano J.J. (2015). Variation in the microbiota of *Ixodes* ticks with regard to geography, species, and sex. Appl Environ Microbiol.

[95] Swei A., Kwan J.Y. (2017). Tick microbiome and pathogen acquisition altered by host blood meal. ISME J.

[96] Takano A., Fujita H., Kadosaka T., Takahashi M., Yamauchi T., Ishiguro F. (2014). Construction of a DNA database for ticks collected in Japan: application of molecular identification based on the mitochondrial 16S rDNA gene. Med Entomol Zool.

[97] Han S.-W., Chae J.-B., Jo Y.-S., Cho Y.-K., Kang J.-G., Shin N.-S. (2021). First detection of *Borrelia* and *Rickettsia* species from *Ornithodoros* ticks in the Republic of Korea. Ticks Tick-Borne Dis.

[98] Costa G.C., Soares A.C., Pereira M.H., Gontijo N.F., Sant’Anna M.R., Araujo R.N. (2015). Life cycle of *Ornithodoros rostratus* (Acari: Argasidae) ticks feeding on mice under laboratory conditions. Exp Appl Acarol.

[99] Smith T.A., Driscoll T., Gillespie J.J., Raghavan R. (2015). A *Coxiella*-like endosymbiont is a potential vitamin source for the Lone Star tick. Genome Biol Evol.

[100] Gerhart J.G., Auguste Dutcher H., Brenner A.E., Moses A.S., Grubhoffer L., Raghavan R. (2018). Multiple acquisitions of pathogen-derived *Francisella* endosymbionts in soft ticks. Genome Biol Evol.

[101] Duron O., Morel O., Noël V., Buysse M., Binetruy F., Lancelot R. (2018). Tick-bacteria mutualism depends on B vitamin synthesis pathways. Curr Biol.

[102] Buysse M., Duron O. (2020). Two novel *Rickettsia* species of soft ticks in North Africa: *’Candidatus* Rickettsia africaseptentrionalis’ and *’Candidatus* Rickettsia mauretanica’. Ticks Tick-Borne Dis.

[103] Lado P., Luan B., Allerdice M.E.J., Paddock C.D., Karpathy S.E., Klompen H. Integrating population genetic structure, microbiome, and pathogens presence data in *Dermacentor variabilis*. PeerJ 2020;8:e9367-e9367. 10.7717/peerj.9367 PMC7350919 32704442

[104] Stathopoulos S., Neafsey D.E., Lawniczak M.K., Muskavitch M.A., Christophides G.K. (2014). Genetic dissection of *Anopheles gambiae* gut epithelial responses to *Serratia marcescens*. PLoS Pathog.

[105] Egan S.L., Taylor C.L., Banks P.B., Northover A.S., Ahlstrom L.A., Ryan U.M. (2021). The bacterial biome of ticks and their wildlife hosts at the urban–wildland interface. Microb Genomics.

[106] Douglas A.E. (2016). How multi-partner endosymbioses function. Nat Rev Microbiol.

[107] Buysse M., Floriano A.M., Gottlieb Y., Nardi T., Comandatore F., Olivieri E. (2021). A dual endosymbiosis supports nutritional adaptation to hematophagy in the invasive tick *Hyalomma marginatum*. Elife.

[108] Wu D., Daugherty S.C., Van Aken S.E., Pai G.H., Watkins K.L., Khouri H. (2006). Metabolic complementarity and genomics of the dual bacterial symbiosis of sharpshooters. PLoS Biol.

[109] Oliver K.M., Degnan P.H., Hunter M.S., Moran N.A. (2009). Bacteriophages encode factors required for protection in a symbiotic mutualism. Science.

[110] Špitalská E., Sparagano O., Stanko M., Schwarzová K., Špitalský Z., Škultéty Ľ. (2018). Diversity of *Coxiella*-like and *Francisella*-like endosymbionts, and *Rickettsia* spp., *Coxiella burnetii* as pathogens in the tick populations of Slovakia, Central Europe. Ticks Tick-Borne Dis.

[111] Gerhart J.G., Moses A.S., Raghavan R. (2016). A *Francisella*-like endosymbiont in the Gulf Coast tick evolved from a mammalian pathogen. Sci Rep.

[112] Scanes C.G., Scanes C.G. (2015). Sturkie's Avian physiology (Sixth Edition).

[113] Mediannikov O., Nguyen T.-T., Bell-Sakyi L., Padmanabhan R., Fournier P.-E., Raoult D. (2014). High quality draft genome sequence and description of *Occidentia massiliensis* gen. nov., sp. nov., a new member of the family *Rickettsiaceae*. Stand Gen Sci.

[114] Hornok S., Kontschán J., Takács N., Chaber A.L., Halajian A., Szekeres S. (2021). *Rickettsiaceae* in two reptile-associated tick species, *Amblyomma exornatum* and *Africaniella transversale*: First evidence of *Occidentia massiliensis* in hard ticks (Acari: Ixodidae). Ticks Tick-Borne Dis.

[115] Lucas M., Sur une nouvelle espèce d’Arachnide qui appartient au genre Ixodes , et qui vit dans le contour interne de la cavité orbitaire de Python sebae, Duméril et Bibron (Coluber sebae, Gmelin), Annales de la Société Entomologique de France, Série. 1845; pp. 61-65.

[116] Sylla M., Pourrut X., Faye N., Ba K., Cornet J.-P., Camicas J.-L. (2004). Argasidae (Acari: Ixodida) parasites of wild and domestic animals in Senegal: 1-Review and distribution. Acarologia.

[117] Rossitto De Marchi B., Smith H.A. (2020). Bacterial endosymbiont diversity among *Bemisia tabaci* (Hemiptera: Aleyrodidae) populations in Florida. Insects.

[118] Li T., Xiao J.-H., Xu Z.-H., Murphy R.W., Huang D.-W. (2011). A possibly new *Rickettsia*-like genus symbiont is found in Chinese wheat pest aphid, *Sitobion miscanthi* (Hemiptera: Aphididae). J Invertebr Pathol.

[119] Liu X.-D., Lei H.-X., Chen F.-F. (2019). Infection pattern and negative effects of a facultative endosymbiont on its insect host are environment-dependent. Sci Rep.

[120] Palomar A.M., Veiga J., Portillo A., Santibáñez S., Václav R., Santibáñez P. (2021). Novel genotypes of nidicolous *Argas* ticks and their associated microorganisms from Spain. Front Vet Sci.

[121] Zolnik C.P., Prill R.J., Falco R.C., Daniels T.J., Kolokotronis S.-O. (2016). Microbiome changes through ontogeny of a tick pathogen vector. Mol Ecol.

[122] Gil J.C., Helal Z.H., Risatti G., Hird S.M. (2020). Ixodes scapularis microbiome correlates with life stage, not the presence of human pathogens, in ticks submitted for diagnostic testing. PeerJ.

[123] Kwan J.Y., Griggs R., Chicana B., Miller C., Swei A. (2017). Vertical vs. horizontal transmission of the microbiome in a key disease vector, *Ixodes pacificus*. Mol Ecol.

[124] Gerhart J.G., Moses A.S., Raghavan R. (2016). A *Francisella*-like endosymbiont in the Gulf Coast tick evolved from a mammalian pathogen. Sci Rep.

[125] Williams-Newkirk A.J., Rowe L.A., Mixson-Hayden T.R., Dasch G.A. Characterization of the bacterial communities of life stages of free living lone star ticks (*Amblyomma americanum*). PLoS One 2014;9(7):e102130-e102130. 10.1371/journal.pone.0102130 PMC4108322 25054227

[126] Kagemann J., Clay K. (2013). Effects of infection by *Arsenophonus* and *Rickettsia* bacteria on the locomotive ability of the ticks *Amblyomma americanum, Dermacentor variabilis*, and *Ixodes scapularis*. J Med Entomol.

[127] Gómez-Díaz E., Morris-Pocock J.A., González-Solís J., McCoy K.D. (2012). Trans-oceanic host dispersal explains high seabird tick diversity on Cape Verde islands. Biol Lett.

[128] Taira M., Ando S., Kawabata H., Fujita H., Kadosaka T., Sato H. (2019). Isolation and molecular detection of *Ehrlichia* species from ticks in western, central, and eastern Japan. Ticks Tick-Borne Dis.

[129] Qiu Y., Kidera N., Hayashi M., Fujishima K., Tamura H. (2021). *Rickettsia* spp. and *Ehrlichia* spp. in *Amblyomma* ticks parasitizing wild amphibious sea kraits and yellow-margined box turtles in Okinawa, Japan. Ticks Tick-Borne Dis.

[130] Su H., Onoda E., Tai H., Fujita H., Sakabe S., Azuma K. (2021). Diversity unearthed by the estimated molecular phylogeny and ecologically quantitative characteristics of uncultured *Ehrlichia* bacteria in *Haemaphysalis* ticks, Japan. Sci Rep.

[131] Socolovschi C., Kernif T., Raoult D., Parola P. (2012). *Borrelia*, *Rickettsia*, and *Ehrlichia* species in bat ticks, France, 2010. Emerg Infect Dis.

[132] Yamaji K., Aonuma H., Kanuka H. (2018). Distribution of tick-borne diseases in Japan: Past patterns and implications for the future. J Infect Chemother.

[133] Nakayama S., Kobayashi T., Nakamura A., Yoshitomi H., Song Y., Ashizuka Y. (2020). Detection of *Borrelia* DNA in tick species collected from vegetation and wild animals in Fukuoka, Japan. Japan J Infect Dis.

[134] Taylor K.R., Takano A., Konnai S., Shimozuru M., Kawabata H., Tsubota T. (2012). *Borrelia miyamotoi* infections among wild rodents show age and month independence and correlation with *Ixodes persulcatus* larval attachment in Hokkaido, Japan. Vector-Borne Zoo Dise.

[135] Takano A., Muto M., Sakata A., Ogasawara Y., Ando S., Hanaoka N. (2009). Relapsing fever spirochete in seabird tick, Japan. Emerg Infect Dis.

[136] Lafri I., El Hamzaoui B., Bitam I., Leulmi H., Lalout R., Mediannikov O. (2017). Detection of relapsing fever *Borrelia* spp., *Bartonella* spp., and *Anaplasmataceae* bacteria in argasid ticks in Algeria. PLoS Negl Trop Dis.

[137] Michalski M.L., Bain O., Fischer K., Fischer P.U., Kumar S., Foster J.M. (2010). Identification and phylogenetic analysis of *Dirofilaria ursi* (Nematoda: Filarioidea) from Wisconsin black bears (*Ursus americanus*) and its *Wolbachia* endosymbiont. J Parasitol.

[138] Manoj R.R.S., Latrofa M.S., Epis S., Otranto D. (2021). Wolbachia: endosymbiont of onchocercid nematodes and their vectors. Parasites Vectors.

[139] Correa C.C., Ballard J.W.O. (2016). *Wolbachia* Associations with Insects: Winning or losing against a master manipulator. Front Ecol Evol.

[140] Luu L., Palomar A.M., Farrington G., Schilling A.-K., Premchand-Branker S., McGarry J. (2021). Bacterial pathogens and symbionts harboured by *Ixodes ricinus* ticks parasitising red squirrels in the United Kingdom. Pathogens.

[141] Lejal E., Chiquet J., Aubert J., Robin S., Estrada-Peña A., Rue O. (2021). Temporal patterns in *Ixodes ricinus* microbial communities: an insight into tick-borne microbe interactions. Microbiome.

[142] Plantard O., Bouju-Albert A., Malard M.-A., Hermouet A., Capron G., Verheyden H. (2012). Detection of *Wolbachia* in the tick *Ixodes ricinus* is due to the presence of the hymenoptera endoparasitoid *Ixodiphagus hookeri*. PLoS ONE.

