# Supplementary material for: Novel symbionts and potential human pathogens excavated from argasid tick microbiomes that are shaped by dual or single symbiosis
Source: Comput Struct Biotechnol J. 2022 Apr 19;20:1979–92. doi: 10.1016/j.csbj.2022.04.020 (PMC9062450; doi:10.1016/j.csbj.2022.04.020)
Supplement: Supplementary data 1 [file mmc1.pdf]

Novel symbionts and potential human pathogens excavated from argasid tick microbiomes that are shaped by dual or single symbiosis

Mohamed Abdallah Mohamed Moustafa[a,b], Wessam Mohamed Ahmed Mohamed[a,c], Alice CC Lau[d], Elisha Chatanga[a,e], Yongjin Qiu[f], Naoki Hayashi[a], Doaa Naguib [a,g], Kozue Sato[h], Ai Takano[i], Keita Matsuno[j,k,l], Nariaki Nonaka[a], DeMar Taylor[m], Hiroki Kawabata[n], Ryo Nakao[a,*]

[a] Laboratory of Parasitology, Department of Disease Control, Faculty of Veterinary Medicine, Hokkaido University, Sapporo, Hokkaido 060-0818, Japan.
[b] Department of Animal Medicine, Faculty of Veterinary Medicine, South Valley University, Qena 83523, Egypt.
[c] Division of Bioinformatics, Research Center for Zoonosis Control, Hokkaido University, Sapporo, Hokkaido 001-0020, Japan.
[d] Laboratory of Wildlife Biology and Medicine, Department of Environmental Veterinary Sciences, Faculty of Veterinary Medicine, Hokkaido University, Sapporo, Hokkaido 060-0818, Japan.
[e] Department of Veterinary Pathobiology, Faculty of Veterinary Medicine, Lilongwe University of Agriculture and Natural Resources. P.O. Box 219, Lilongwe, Malawi.
[f] Division of International Research Promotion, International Institute for Zoonosis Control, Hokkaido University, Sapporo, Hokkaido 001-0020, Japan.
[g] Department of Hygiene and Zoonoses, Faculty of Veterinary Medicine, Mansoura University, Mansoura 35516, Egypt.
[h] Laboratory of Systemic Infection, Department of Bacteriology-I National Institute of Infectious Diseases Toyama 1-23-1, Shinjuku-ku, Tokyo 162-8640 Japan.
[i] Laboratory of Epidemiology, Department of Veterinary Medicine, Joint Faculty of Veterinary Medicine, Yamaguchi University 1677-1 Yoshida, Yamaguchi 753-8515, Japan.
[j] Division of Risk Analysis and Management, International Institute for Zoonosis Control, Hokkaido University, Sapporo, Hokkaido 001-0020, Japan.
[k] One Health Research Center, Hokkaido University, Sapporo, Hokkaido, 001-0020, Japan.
[l] International Collaboration Unit, International Institute for Zoonosis Control, Hokkaido University, Sapporo, Hokkaido 001-0020, Japan.
[m] Faculty of Life and Environmental Sciences, University of Tsukuba, Tsukuba, Ibaraki 305-8572, Japan.
[n] Department of Bacteriology-I, National Institute of Infectious Diseases, Toyama 1-23-1, Shinjuku-ku, Tokyo 162-8640, Japan.

[*] **Corresponding author**
Ryo Nakao: ryo.nakao@vetmed.hokudai.ac.jp
Laboratory of Parasitology, Department of Disease Control, Faculty of Veterinary Medicine, Hokkaido University, Sapporo, Hokkaido 060-0818, Japan.

Supplementary Tables:

Table S1. Tick samples used in this study.

	A. japonicus	***C. vespertilionis***	***O. capensis***	***O. sawaii***	***O. moubata***
**Collection site (GPS)**	35.54 N 138.90 E	36.23 N 137.97 E	28.05 N 129.19 E	35.72 N 135.44 E	NA
**Nymph**	4	8	17	6	5
**Male**	14	0	18	8	5
**Female**	20	6	14	7	5
**Total**	38	14	49	21	15

NA, not applicable.

Table S2. Generalized linear model output for Shannon diversity comparing argasid tick species and sex/stage.

glm (Exp_Shannon~ Species+Stage/sex, data = All_tick_species)				
**Variable**	**Estimate**	**Std. Error**	**t ratio**	***p* value**
A. japonicus* - *C. vespertilionis	-44.68	40.58	-1.10	0.81
A. japonicus* - *O. capensis	-256.54	27.58	-9.30	**0.00**
A. japonicus* - *O. sawaii	-99.51	34.70	-2.87	**0.04**
A. japonicus* - *O. moubata	-26.89	38.24	-0.70	0.96
C. vespertilionis* - *O. capensis	-211.86	38.56	-5.49	**0.00**
C. vespertilionis* - *O. sawaii	-54.83	44.27	-1.24	0.73
C. vespertilionis* - *O. moubata	17.79	46.78	0.38	1.00
O. capensis* - *O. sawaii	157.03	32.90	4.77	**0.00**
O. capensis* - *O. moubata	229.65	36.58	6.28	**0.00**
O. sawaii* - *O. moubata	72.62	42.36	1.71	0.43
**Female - Male**	38.35	25.87	1.48	0.30
**Female - Nymph**	122.69	27.21	4.51	**0.00**
**Male - Nymph**	84.34	28.18	2.99	**0.01**

Significant values are written in bold red fonts ($p < 0.05$).

Table S3. Generalized linear model output for Faith's phylogenetic diversity comparing argasid tick species and sex/stage.

glm (Log_Faith's_PD~ Species+Stage/sex, data = All_tick_species)				
**Variable**	**Estimate**	**Std. Error**	**t value**	***p* value**
A. japonicus* - *C. vespertilionis	0.39	0.16	2.52	0.09
A. japonicus* - *O. capensis	-0.73	0.11	-6.80	**0.00**
A. japonicus* - *O. sawaii	-0.42	0.13	-3.13	**0.02**
A. japonicus* - *O. moubata	1.19	0.15	8.03	**0.00**
C. vespertilionis* - *O. capensis	-1.12	0.15	-7.51	**0.00**
C. vespertilionis* - *O. sawaii	-0.81	0.17	-4.76	**0.00**
C. vespertilionis* - *O. moubata	0.79	0.18	4.38	**0.00**
O. capensis* - *O. sawaii	0.30	0.13	2.40	0.12
O. capensis* - *O. moubata	1.91	0.14	13.52	**0.00**
O. sawaii* - *O. moubata	1.61	0.16	9.82	**0.00**
**Female - Male**	0.11	0.10	1.08	0.53
**Female - Nymph**	0.49	0.11	4.63	**0.00**
**Male - Nymph**	0.38	0.11	3.48	**0.00**

Significant values are written in bold red fonts ($p < 0.05$).

Table S4. Generalized linear model output for observed OTUs comparing argasid tick species and sex/stage.

glm (Log_Observed_OTUs~ Species+Stage/sex, data = All_tick_species)				
**Variable**	**Estimate**	**Std. Error**	**t value**	***p* value**
A. japonicus* - *C. vespertilionis	-0.23	0.15	-1.55	0.53
A. japonicus* - *O. capensis	-2.00	0.10	-20.05	**0.00**
A. japonicus* - *O. sawaii	-1.37	0.13	-10.91	**0.00**
A. japonicus* - *O. moubata	0.96	0.14	6.90	**0.00**
C. vespertilionis* - *O. capensis	-1.77	0.14	-12.70	**0.00**
C. vespertilionis* - *O. sawaii	-1.14	0.16	-7.13	**0.00**
C. vespertilionis* - *O. moubata	1.18	0.17	6.99	**0.00**
O. capensis* - *O. sawaii	0.63	0.12	5.29	**0.00**
O. capensis* - *O. moubata	2.96	0.13	22.33	**0.00**
O. sawaii* - *O. moubata	2.33	0.15	15.17	**0.00**
**Female - Male**	-0.02	0.09	-0.19	0.98
**Female - Nymph**	0.50	0.10	5.11	**0.00**
**Male - Nymph**	0.52	0.10	5.10	**0.00**

Significant values are written in bold red fonts ($p < 0.05$).

Table S5. Generalized linear model output for Pielou's evenness comparing argasid tick species and sex/stage.

glm (Log_Evenness~ Species+Stage/sex, data = All_tick_species)				
**Variable**	**Estimate**	**Std. Error**	**t value**	***p* value**
A. japonicus* - *C. vespertilionis	-0.72	0.13	-5.75	**0.00**
A. japonicus* - *O. capensis	-1.57	0.09	-18.43	**0.00**
A. japonicus* - *O. sawaii	-1.49	0.11	-13.90	**0.00**
A. japonicus* - *O. moubata	-0.81	0.12	-6.81	**0.00**
C. vespertilionis* - *O. capensis	-0.85	0.12	-7.13	**0.00**
C. vespertilionis* - *O. sawaii	-0.77	0.14	-5.62	**0.00**
C. vespertilionis* - *O. moubata	-0.08	0.14	-0.58	0.98
O. capensis* - *O. sawaii	0.08	0.10	0.79	0.93
O. capensis* - *O. moubata	0.77	0.11	6.77	**0.00**
O. sawaii* - *O. moubata	0.69	0.13	5.24	**0.00**
**Female - Male**	-0.16	0.08	-1.99	0.12
**Female - Nymph**	-0.13	0.08	-1.58	0.26
**Male - Nymph**	0.03	0.09	0.30	0.95

Significant values are written in bold red fonts ($p < 0.05$).

Table S6. Generalized linear model output for all alpha diversity metrics comparing the effect of sex/stage in each argasid tick species.

glm (Alpha_diversity ~ Stage/sex , data = Each_tick_species)						
**Species**	**Alpha Diversity**	**Variable**	**Estimate**	**Std. Error**	**t value**	***p* value**
A. japonicus	Shannon	Female - Male	-1.11	0.59	-1.89	0.16
		Female - Nymph	-1.26	0.92	-1.36	0.37
		Male - Nymph	-0.14	0.96	-0.15	0.99
	Faith's PD	Female - Male	0.14	0.20	0.68	0.78
		Female - Nymph	0.24	0.32	0.77	0.72
		Male - Nymph	0.11	0.33	0.33	0.94
	Observed OTUs	Female - Male	-0.19	0.09	-2.09	0.11
		Female - Nymph	-0.07	0.14	-0.50	0.87
		Male - Nymph	0.12	0.15	0.80	0.70
	Pielou's evenness	Female - Male	-0.45	0.22	-2.08	0.11
		Female - Nymph	-0.81	0.34	-2.39	0.06
		Male - Nymph	-0.36	0.35	-1.03	0.56
C. vespertilionis	Shannon	Female - Nymph	-1.47	0.87	-1.70	0.12
	Faith's PD	Female - Nymph	-0.15	0.19	-0.75	0.47
	Observed OTUs	Female - Nymph	0.03	0.13	0.22	0.83
	Pielou's evenness	Female - Nymph	-0.29	0.13	-2.12	0.06
O. capensis	Shannon	Female - Male	134.83	64.42	2.09	0.10
		Female - Nymph	332.18	65.24	5.09	**0.00**
		Male - Nymph	197.35	61.14	3.23	**0.01**
	Faith's PD	Female - Male	0.16	0.14	1.12	0.51
		Female - Nymph	0.92	0.14	6.54	**0.00**
		Male - Nymph	0.77	0.13	5.80	**0.00**
	Observed OTUs	Female - Male	0.31	0.17	1.83	0.17
		Female - Nymph	1.22	0.17	7.17	**0.00**
		Male - Nymph	0.91	0.16	5.73	**0.00**
	Pielou's evenness	Female - Male	0.069	0.040	1.74	0.20
		Female - Nymph	0.108	0.041	2.66	**0.03**
		Male - Nymph	0.039	0.038	1.01	0.57
O. sawaii	Shannon	Female - Male	47.00	37.49	1.25	0.44
		Female - Nymph	13.29	40.08	0.33	0.94
		Male - Nymph	-33.71	37.49	-0.90	0.65
	Faith's PD	Female - Male	0.44	0.22	2.03	0.14
		Female - Nymph	0.45	0.23	1.96	0.15
		Male - Nymph	0.01	0.22	0.07	1.00
	Observed OTUs	Female - Male	0.41	0.27	1.53	0.30
		Female - Nymph	0.38	0.28	1.34	0.39
		Male - Nymph	-0.02	0.27	-0.09	1.00
	Pielou's evenness	Female - Male	0.04	0.07	0.57	0.84
		Female - Nymph	-0.05	0.08	-0.57	0.84
		Male - Nymph	-0.09	0.07	-1.19	0.48
O. moubata	Shannon	Female - Male	-0.45	0.41	-1.08	0.54
		Female - Nymph	0.48	0.41	1.15	0.50
		Male - Nymph	0.92	0.41	2.23	0.11
	Faith's PD	Female - Male	-0.18	0.15	-1.21	0.47
		Female - Nymph	0.10	0.15	0.68	0.78
		Male - Nymph	0.27	0.15	1.89	0.18
	Observed OTUs	Female - Male	-0.25	0.15	-1.71	0.24
		Female - Nymph	0.16	0.15	1.06	0.55
		Male - Nymph	0.41	0.15	2.77	**0.04**
	Pielou's evenness	Female - Male	-0.09	0.12	-0.73	0.75
		Female - Nymph	0.34	0.12	2.75	**0.04**
		Male - Nymph	0.43	0.12	3.48	**0.01**

Significant values are written in bold red fonts ($p < 0.05$).

Table S7. Adonis PERMANOVA test results for all beta diversity metrics comparing the effect of species and sex/stage in argasid ticks.

	Variable	Df	SumsOfSqs	MeanSqs	F.Model	R2	Pr(>F)
**Unweighted UniFrac**	Species	4	13.573	3.393	18.582	0.354	**0.001**
	Sex/stage	2	1.242	0.621	3.400	0.032	**0.001**
**Weighted UniFrac**	Species	4	4.645	1.161	54.744	0.614	**0.001**
	Sex/stage	2	0.186	0.093	4.375	0.025	**0.002**
**Jaccard**	Species	4	15.671	3.918	12.773	0.277	**0.001**
	Sex/stage	2	1.269	0.634	2.068	0.022	**0.001**
**Bray-Curtis**	Species	4	28.423	7.106	36.309	0.518	**0.001**
	Sex/stage	2	1.182	0.591	3.020	0.022	**0.002**

Significant values are written in bold red fonts ($p < 0.05$).

Table S8. Pairwise PERMANOVA results comparing the beta diversity metrics between different argadis tick species.

Dissimilarity Index	Group 1	Group 2	Sample size	Permutations	Pseudo F	*p* value	q value
**Unweighted UniFrac**	*A. japonicus*	*C. vespertilionis*	52	999	8.11	**0.001**	0.001
		O. capensis	87	999	24.18	**0.001**	0.001
		O. moubata	53	999	19.00	**0.001**	0.001
		O. sawaii	58	999	16.32	**0.001**	0.001
	C. vespertilionis	*O. capensis*	63	999	15.38	**0.001**	0.001
		O. moubata	29	999	17.69	**0.001**	0.001
		O. sawaii	34	999	13.00	**0.001**	0.001
	O. capensis	*O. moubata*	64	999	26.24	**0.001**	0.001
		O. sawaii	69	999	6.94	**0.001**	0.001
	O. moubata	*O. sawaii*	35	999	25.92	**0.001**	0.001
**Weighted UniFrac**	*A. japonicus*	*C. vespertilionis*	52	999	14.72	**0.001**	0.001
		O. capensis	87	999	96.40	**0.001**	0.001
		O. moubata	53	999	12.24	**0.001**	0.001
		O. sawaii	58	999	42.02	**0.001**	0.001
	C. vespertilionis	*O. capensis*	63	999	57.17	**0.001**	0.001
		O. moubata	29	999	55.26	**0.001**	0.001
		O. sawaii	34	999	36.84	**0.001**	0.001
	O. capensis	*O. moubata*	64	999	54.30	**0.001**	0.001
		O. sawaii	69	999	43.37	**0.001**	0.001
	O. moubata	*O. sawaii*	35	999	48.54	**0.001**	0.001
**Jaccard**	*A. japonicus*	*C. vespertilionis*	52	999	7.24	**0.001**	0.001
		O. capensis	87	999	16.97	**0.001**	0.001
		O. moubata	53	999	21.14	**0.001**	0.001
		O. sawaii	58	999	10.55	**0.001**	0.001
	C. vespertilionis	*O. capensis*	63	999	9.55	**0.001**	0.001
		O. moubata	29	999	18.32	**0.001**	0.001
		O. sawaii	34	999	7.27	**0.001**	0.001
	O. capensis	*O. moubata*	64	999	15.91	**0.001**	0.001
		O. sawaii	69	999	7.81	**0.001**	0.001
	O. moubata	*O. sawaii*	35	999	13.91	**0.001**	0.001
**Bray-Curtis**	*A. japonicus*	*C. vespertilionis*	52	999	30.40	**0.001**	0.001
		O. capensis	87	999	37.96	**0.001**	0.001
		O. moubata	53	999	39.61	**0.001**	0.001
		O. sawaii	58	999	28.66	**0.001**	0.001
	C. vespertilionis	*O. capensis*	63	999	30.61	**0.001**	0.001
		O. moubata	29	999	120.36	**0.001**	0.001
		O. sawaii	34	999	37.67	**0.001**	0.001
	O. capensis	*O. moubata*	64	999	42.12	**0.001**	0.001
		O. sawaii	69	999	21.39	**0.001**	0.001
	O. moubata	*O. sawaii*	35	999	57.66	**0.001**	0.001

Significant values are written in bold red fonts ($p < 0.05$).

Table S9. Pairwise PERMANOVA results for all beta diversity metrics comparing the effect of sex/stage in *A. japonicus*.

Dissimilarity Index	Group 1	Group 2	Sample size	Permutations	Pseudo F	*p* value	q value
**Unweighted UniFrac**	Female	Male	34	999	1.09	0.34	0.376
	Female	Nymph	24	999	1.60	0.148	0.376
	Male	Nymph	18	999	1.01	0.376	0.376
**Weighted UniFrac**	Female	Male	34	999	0.27	0.739	0.739
	Female	Nymph	24	999	0.66	0.368	0.693
	Male	Nymph	18	999	0.76	0.462	0.693
**Jaccard**	Female	Male	34	999	1.83	**0.002**	0.006
	Female	Nymph	24	999	1.24	0.138	0.207
	Male	Nymph	18	999	1.06	0.389	0.389
**Bray-Curtis**	Female	Male	34	999	0.46	0.62	0.62
	Female	Nymph	24	999	0.53	0.372	0.62
	Male	Nymph	18	999	0.60	0.554	0.62

Significant values are written in bold red fonts ($p < 0.05$).

Table S10. Pairwise PERMANOVA results for all beta diversity metrics comparing the effect of sex/stage in *C. vespertilionis*.

Dissimilarity Index	Group 1	Group 2	Sample size	Permutations	Pseudo F	*p* value	q value
**Unweighted UniFrac**	Female	Nymph	14	999	0.93	0.57	0.57
**Weighted UniFrac**	Female	Nymph	14	999	0.95	0.44	0.44
**Jaccard**	Female	Nymph	14	999	1.30	**0.04**	0.04
**Bray-Curtis**	Female	Nymph	14	999	1.02	0.40	0.40

Significant values are written in bold red fonts ($p < 0.05$).

Table S11. Pairwise PERMANOVA results for all beta diversity metrics comparing the effect of sex/stage in *O. capensis*.

Dissimilarity Index	Group 1	Group 2	Sample size	Permutations	Pseudo F	*p* value	q value
**Unweighted UniFrac**	Female	Male	32	999	1.17	0.18	0.18
	Female	Nymph	31	999	6.97	**0.00**	0.00
	Male	Nymph	35	999	6.07	**0.00**	0.00
**Weighted UniFrac**	Female	Male	32	999	0.91	0.44	0.44
	Female	Nymph	31	999	8.72	**0.00**	0.00
	Male	Nymph	35	999	6.05	**0.00**	0.01
**Jaccard**	Female	Male	32	999	1.29	**0.04**	0.04
	Female	Nymph	31	999	3.91	**0.00**	0.00
	Male	Nymph	35	999	3.22	**0.00**	0.00
**Bray-Curtis**	Female	Male	32	999	2.28	**0.01**	0.01
	Female	Nymph	31	999	6.66	**0.00**	0.00
	Male	Nymph	35	999	4.98	**0.00**	0.00

Significant values are written in bold red fonts ($p < 0.05$).

Table S12. Pairwise PERMANOVA results for all beta diversity metrics comparing the effect of sex/stage in *O. sawaii*.

Dissimilarity Index	Group 1	Group 2	Sample size	Permutations	Pseudo F	*p* value	q value
**Unweighted UniFrac**	Female	Male	14	999	1.16	0.23	0.50
	Female	Nymph	12	999	1.08	0.33	0.50
	Male	Nymph	14	999	0.75	0.80	0.80
**Weighted UniFrac**	Female	Male	14	999	0.42	0.94	0.94
	Female	Nymph	12	999	1.09	0.32	0.80
	Male	Nymph	14	999	0.85	0.54	0.80
**Jaccard**	Female	Male	14	999	1.00	0.45	0.70
	Female	Nymph	12	999	0.98	0.55	0.70
	Male	Nymph	14	999	0.91	0.70	0.70
**Bray-Curtis**	Female	Male	14	999	0.92	0.48	0.48
	Female	Nymph	12	999	1.65	0.08	0.25
	Male	Nymph	14	999	1.14	0.28	0.42

Table S13. Pairwise PERMANOVA results for all beta diversity metrics comparing the effect of sex/stage in *O. moubata*.

Dissimilarity Index	Group 1	Group 2	Sample size	Permutations	Pseudo F	*p* value	q value
**Unweighted UniFrac**	Female	Male	10	999	1.65	0.11	0.34
	Female	Nymph	10	999	1.27	0.27	0.41
	Male	Nymph	10	999	0.87	0.54	0.54
**Weighted UniFrac**	Female	Male	10	999	3.59	0.12	0.12
	Female	Nymph	10	999	8.47	**0.04**	0.05
	Male	Nymph	10	999	25.72	**0.01**	0.04
**Jaccard**	Female	Male	10	999	0.75	0.75	0.75
	Female	Nymph	10	999	1.24	0.27	0.41
	Male	Nymph	10	999	1.41	0.12	0.35
**Bray-Curtis**	Female	Male	10	999	3.29	0.12	0.12
	Female	Nymph	10	999	8.46	**0.04**	0.05
	Male	Nymph	10	999	27.38	**0.01**	0.02

Significant values are written in bold red fonts ($p < 0.05$).

Supplementary Figures:

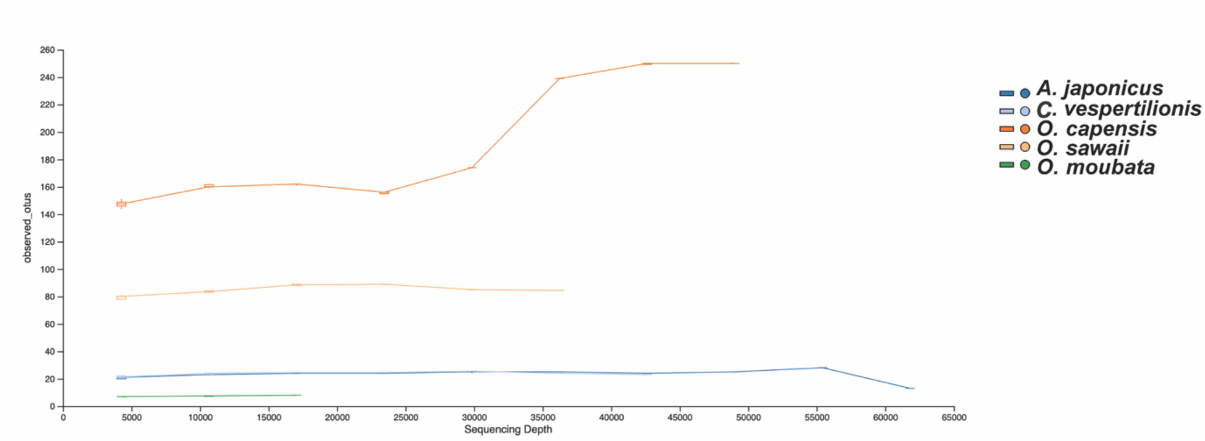

Fig. S1. Rarefaction curves showing the observed species richness in our argasid tick samples.

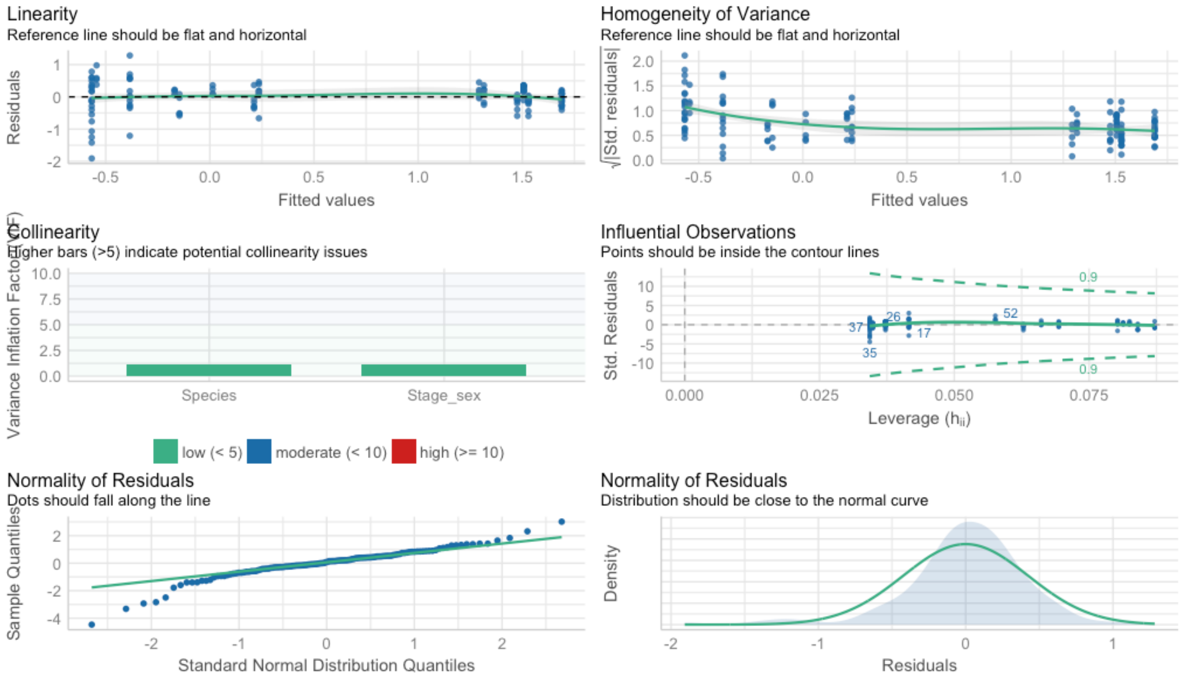

Fig. S2. A model assumption test for Shannon diversity analysis. We checked “glm (Log_Shannon~ Species+Stage/sex, data = All_tick_species)” model by the function “check_model” in the package “performance” in R. The test showed that the linearity, normality, and collinearity are all suitable to use the model with no issues.

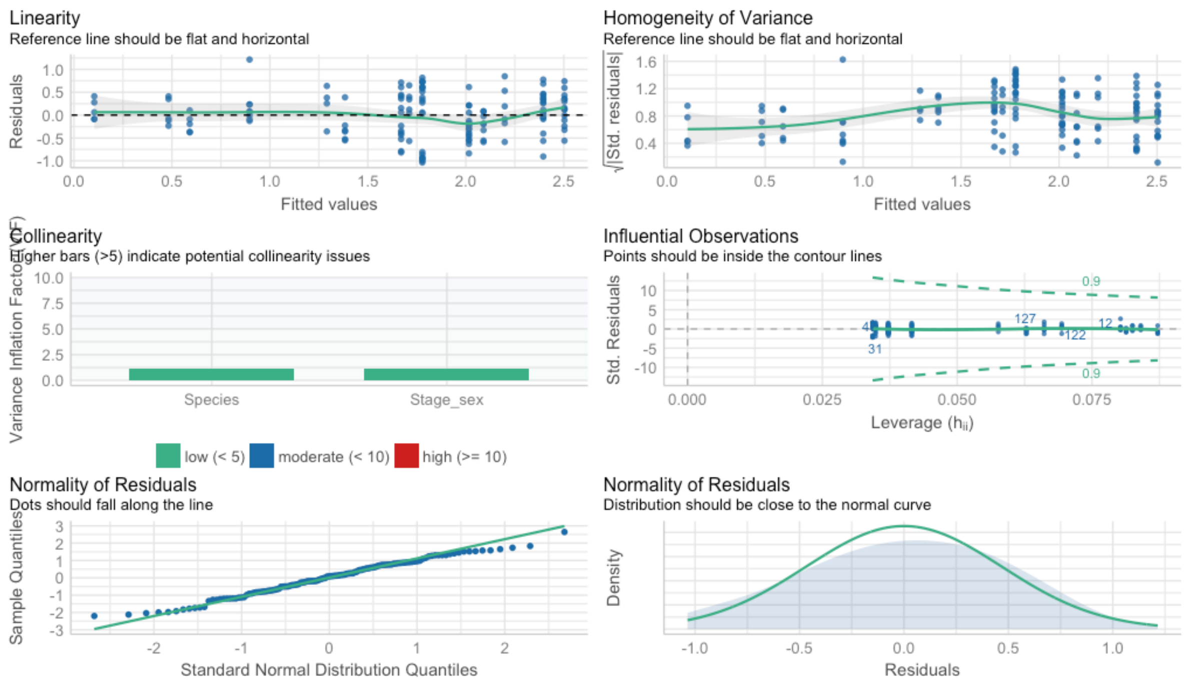

Fig. S3. A model assumption test for Faith's PD analysis. We checked "glm (Log_Faith's_PD~ Species+Stage/sex, data = All_tick_species)" model by the function "check_model" in the package "performance" in R. The test showed that the linearity, normality, and collinearity are all suitable to use the model with no issues.

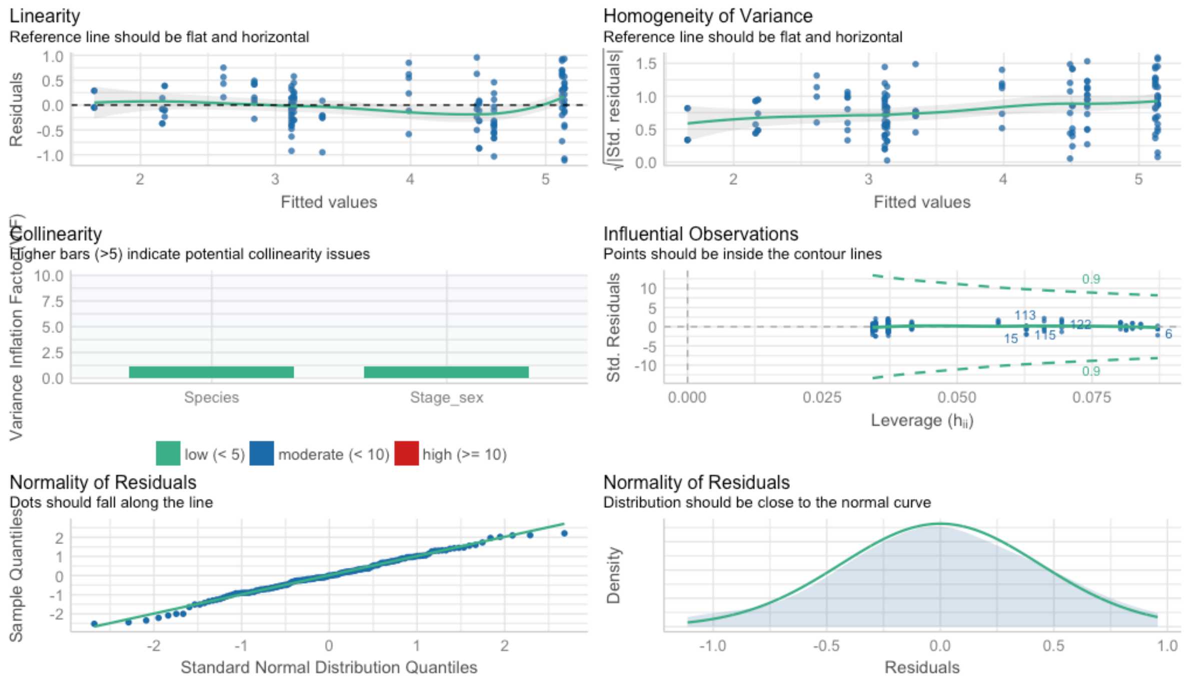

Fig. S4. A model assumption test for observed features analysis. We checked "glm(Log_Observed_Features ~ Species+Stage/sex, data = All_tick_species)" model by the function "check_model" in the package "performance" in R. The linearity, normality, and collinearity are suitable with no issues.

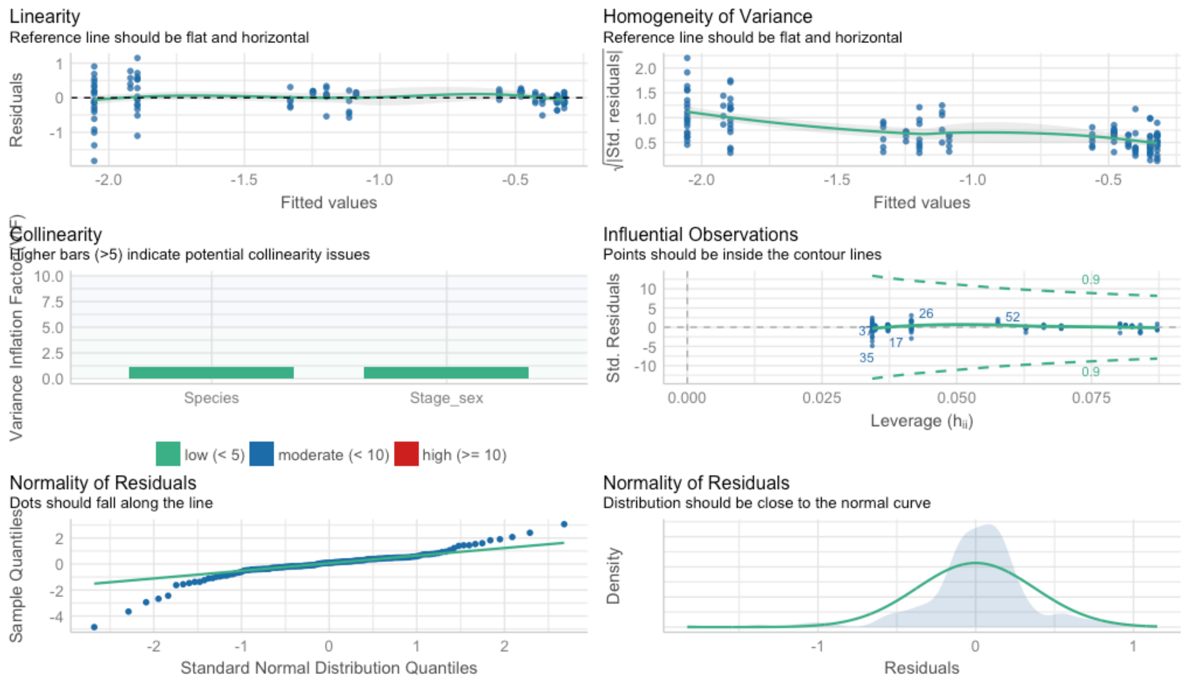

Fig. S5. A model assumption test for Pielou's evenness analysis. We checked "glm (Log_Evennes ~ Species+Stage/sex, data = All_tick_species)" model by the function "check_model" in the package "performance" in R. The linearity, normality, and collinearity are all suitable with no issues.

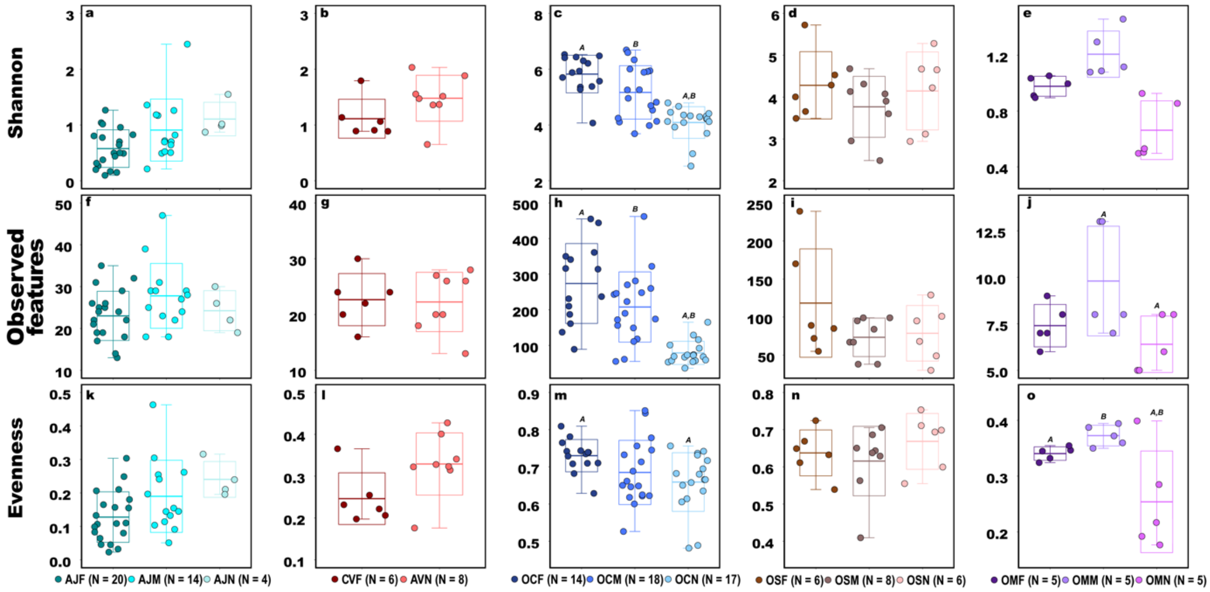

Fig. S6. Alpha diversity analyses of microbial populations from 136 argasid tick samples according to sex and stage variations. Each dot shows the microbial population from an individual argasid tick and color represents sample species, sex, and stage (*A. japonicus* "AJ", *C. vespertilionis* "CV", *O. capensis* "OC", *O. sawaii* "OS", and *O. moubata* "OM"), sex (Female "F" and Male "M" or stage (Nymph "N"). Letters above the bars indicate statistically significant difference (GLM: $p < 0.01$).

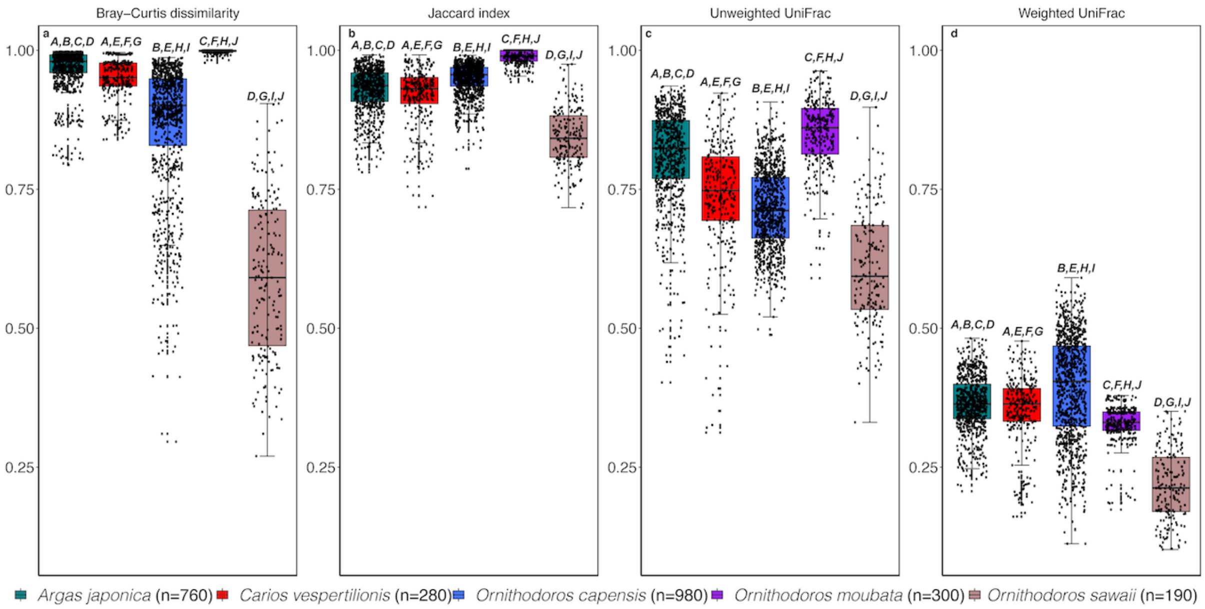

Fig. S7. Beta diversity analyses used to measure the community dissimilarity between different argasid tick species using a pairwise PERMANOVA. Letters above the bars indicate statistically significant difference (GLM: $p < 0.01$).

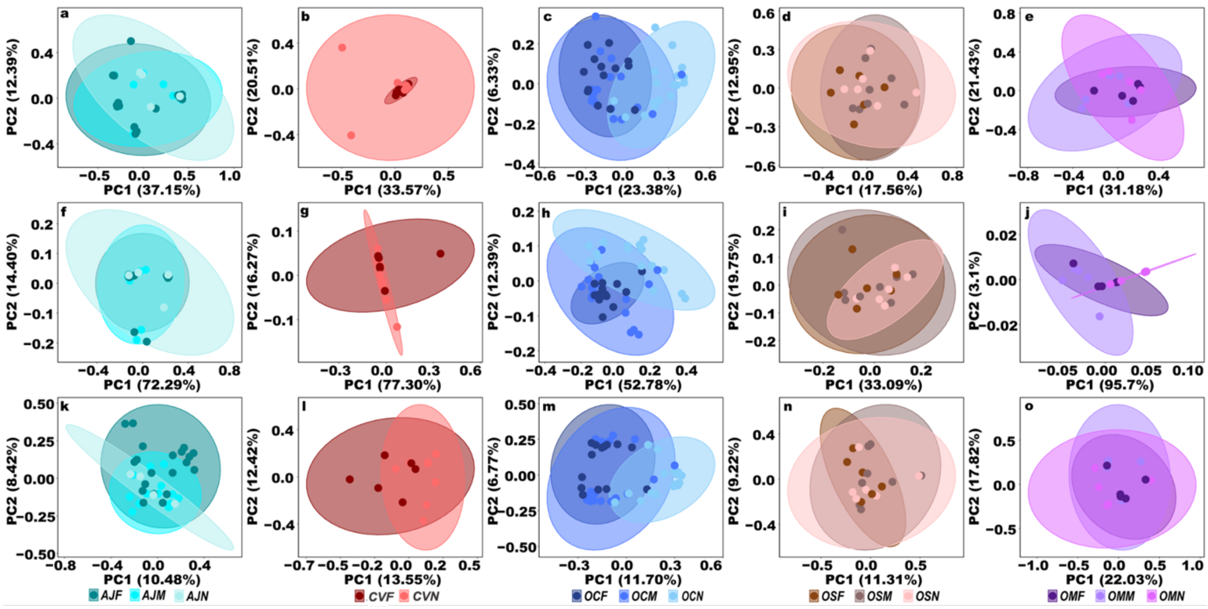

Fig. S8. Beta diversity analyses within argasid ticks. PCoA plots based on: a-e) Unweighted UniFrac f-j) Weighted UniFrac and k-o) Jaccard distances between individual argasid samples. Each dot shows the microbial population from an individual argasid tick and color represents sample species (*A. japonicus* "*AJ*", *C. vespertilionis* "*CV*", *O. capensis* "*OC*", *O. moubata* "*OM*", and *O. sawaii* "*OS*"), sex (Female "*F*" and Male "*M*" or stage (Nymph "*N*").

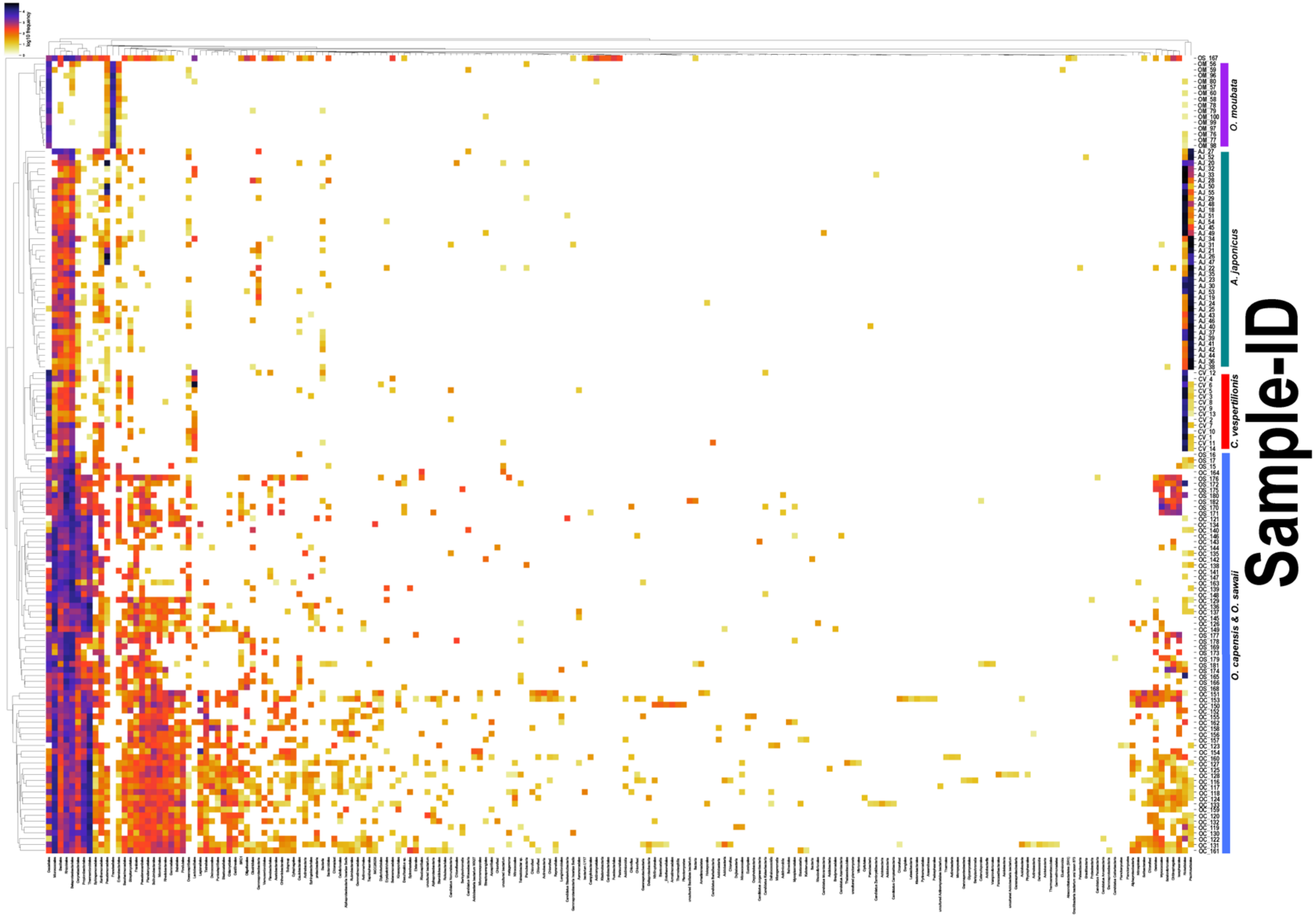

Fig. S9. Maximum abundance heatmaps. Differences in relative abundances of the annotated taxa orders in the microbiome of argasid ticks. Ticks IDs are shown on the right side of the heatmaps. Sample-ID labels show the argasid tick species (*A. japonicus* “AJ”, *C. vespertilionis* “CV”, *O. capensis* “OC”, *O. sawaii* “OS”, and *O. moubata* “OM”) and sample numbers.

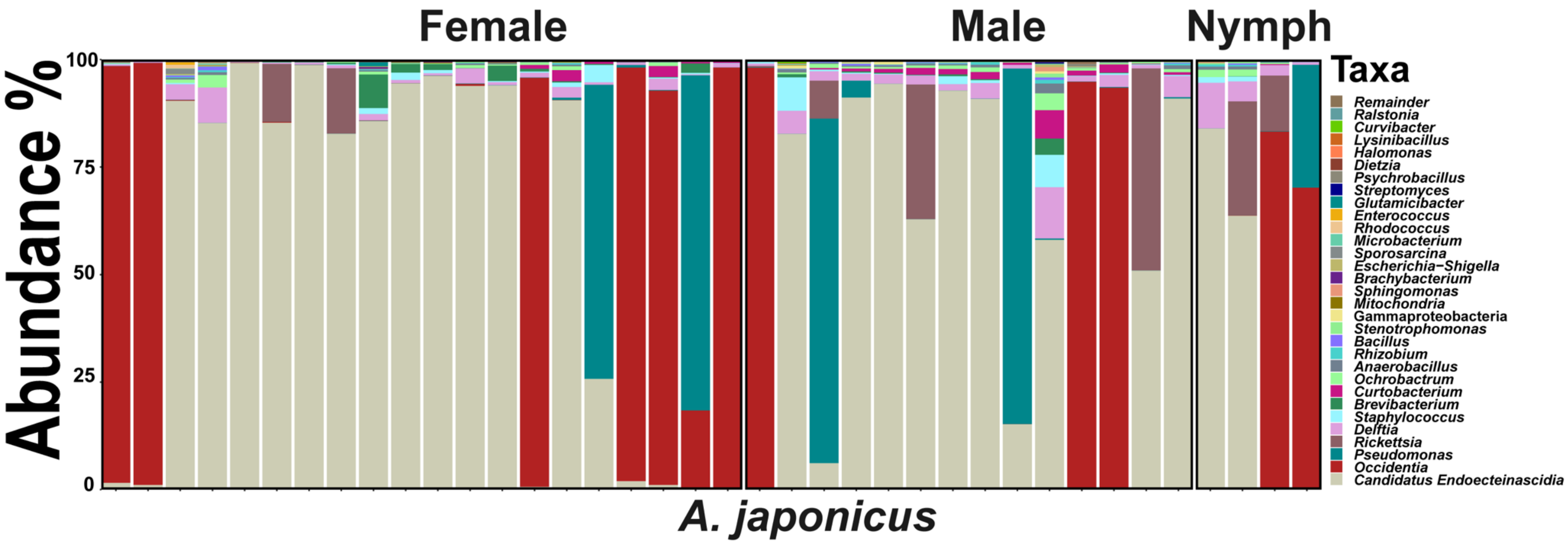

Fig. S10. Relative abundance (%) of bacterial taxa identified in the microbiome of *A. japonicus* samples. The figure displays the highest abundant 30 taxa individually with the remaining grouped together. Each bar represents the bacterial taxa detected in one sample.

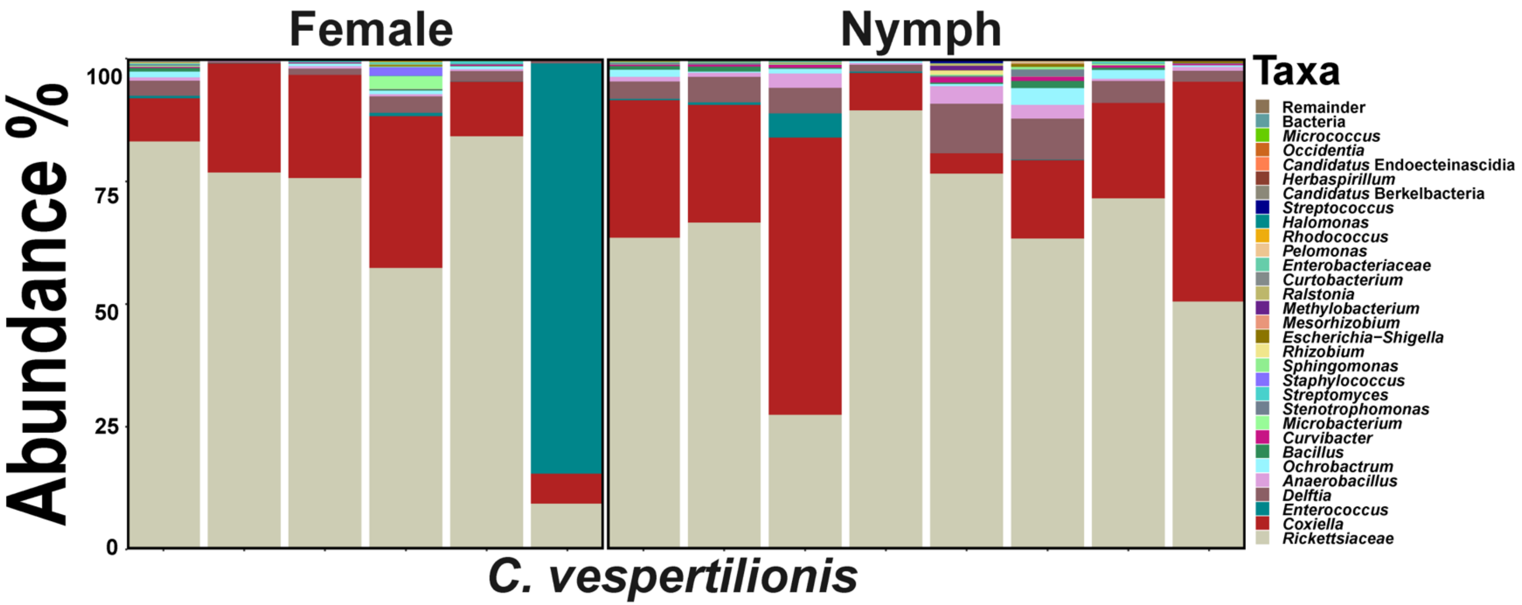

Fig. S11. Relative abundance (%) of bacterial taxa identified in the microbiome of *C. vespertilionis* samples. The figure displays the highest abundant 30 taxa individually with the remaining grouped together. Each bar represents the bacterial taxa detected in one sample.

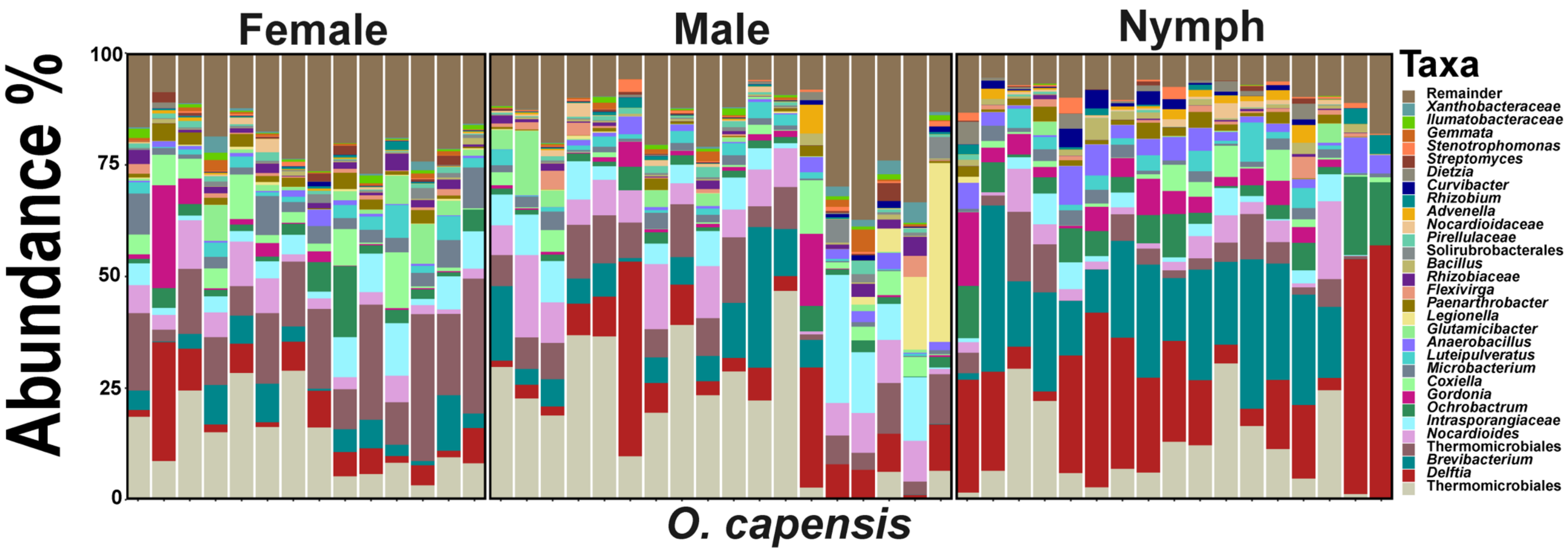

Fig. S12. Relative abundance (%) of bacterial taxa identified in the microbiome of *O. capensis* samples. The figure displays the highest abundant 30 taxa individually with the remaining grouped together. Each bar represents the bacterial taxa detected in one sample.

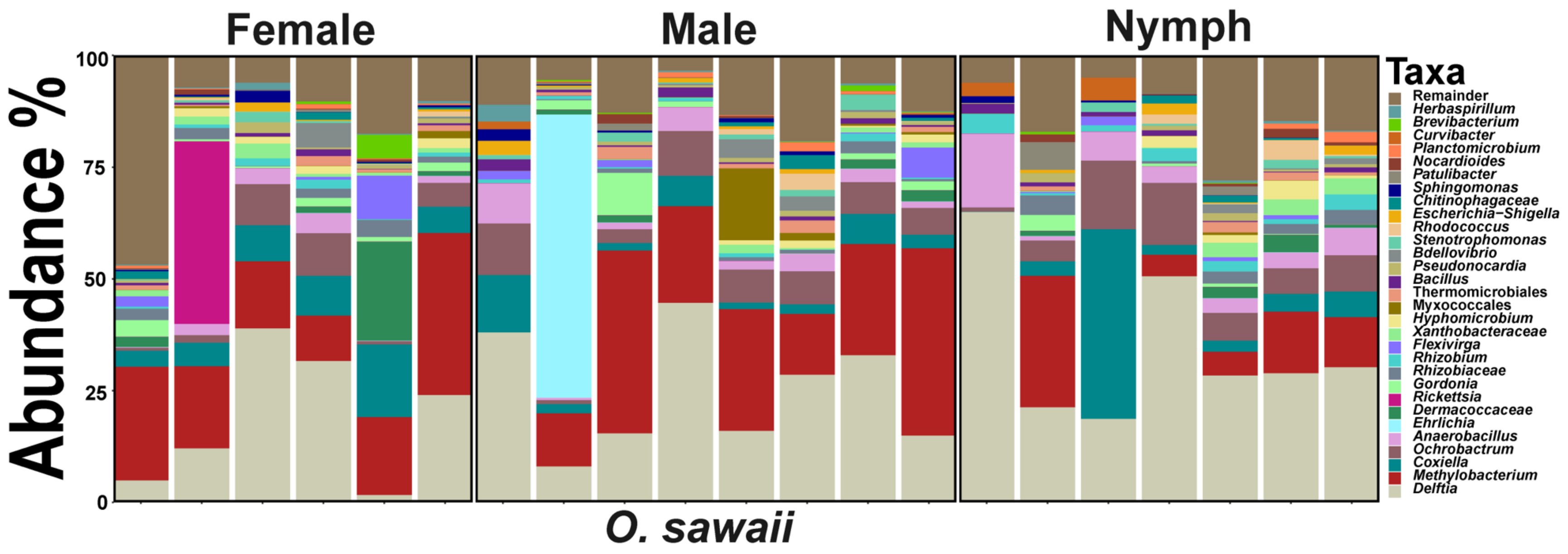

Fig. S13. Relative abundance (%) of bacterial taxa identified in the microbiome of *O. sawaii* samples. The figure displays the highest abundant 30 taxa individually with the remaining grouped together. Each bar represents the bacterial taxa detected in one sample.

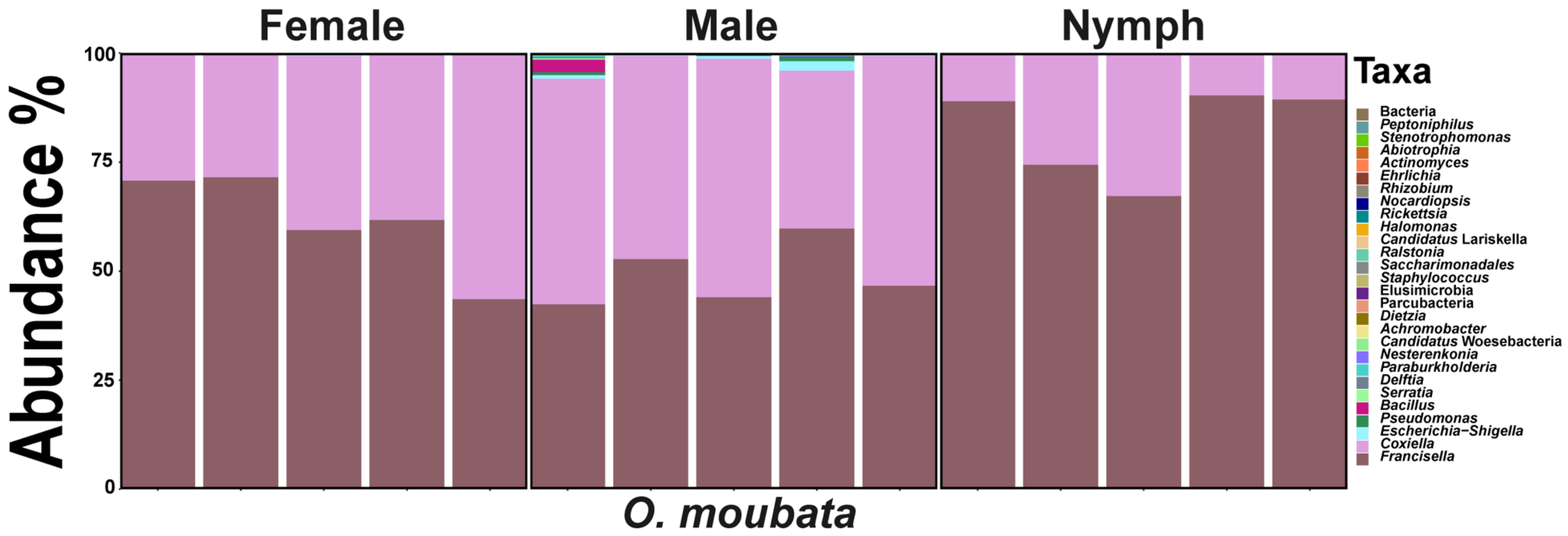

Fig. S14. Relative abundance (%) of bacterial taxa identified in the microbiome of *O. moubata* samples. The figure displays the highest abundant 30 taxa individually with the remaining grouped together. Each bar represents the bacterial taxa detected in one sample.

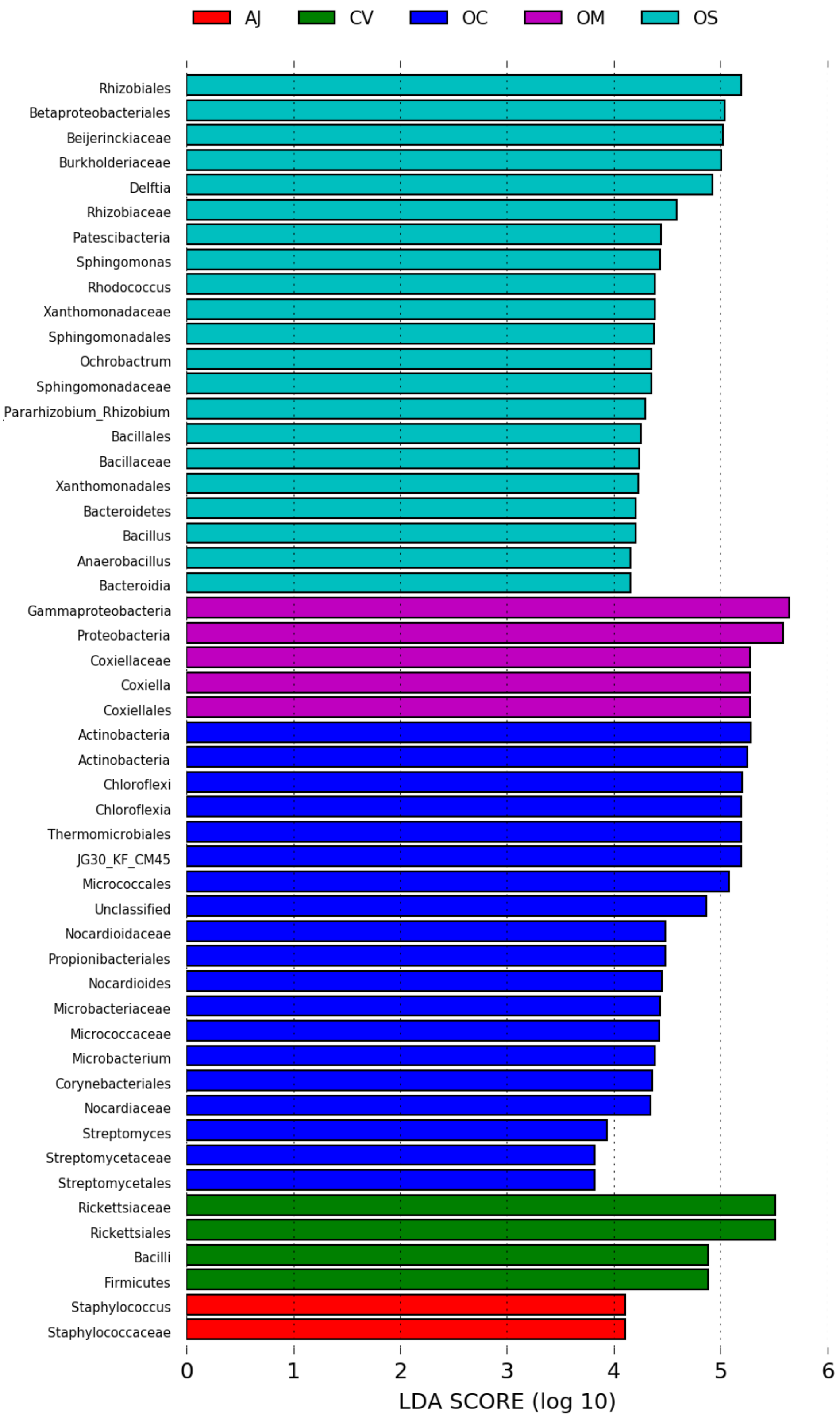

Fig. S15. LEfSe results showing the most differentially abundant taxa ($p < 0.05$) within female argasid ticks and color represents sample species (*A. japonicus* “AJ”, *C. vespertilionis* “CV”, *O. capensis* “OC”, *O. moubata* “OM”, and *O. sawaii* “OS”).

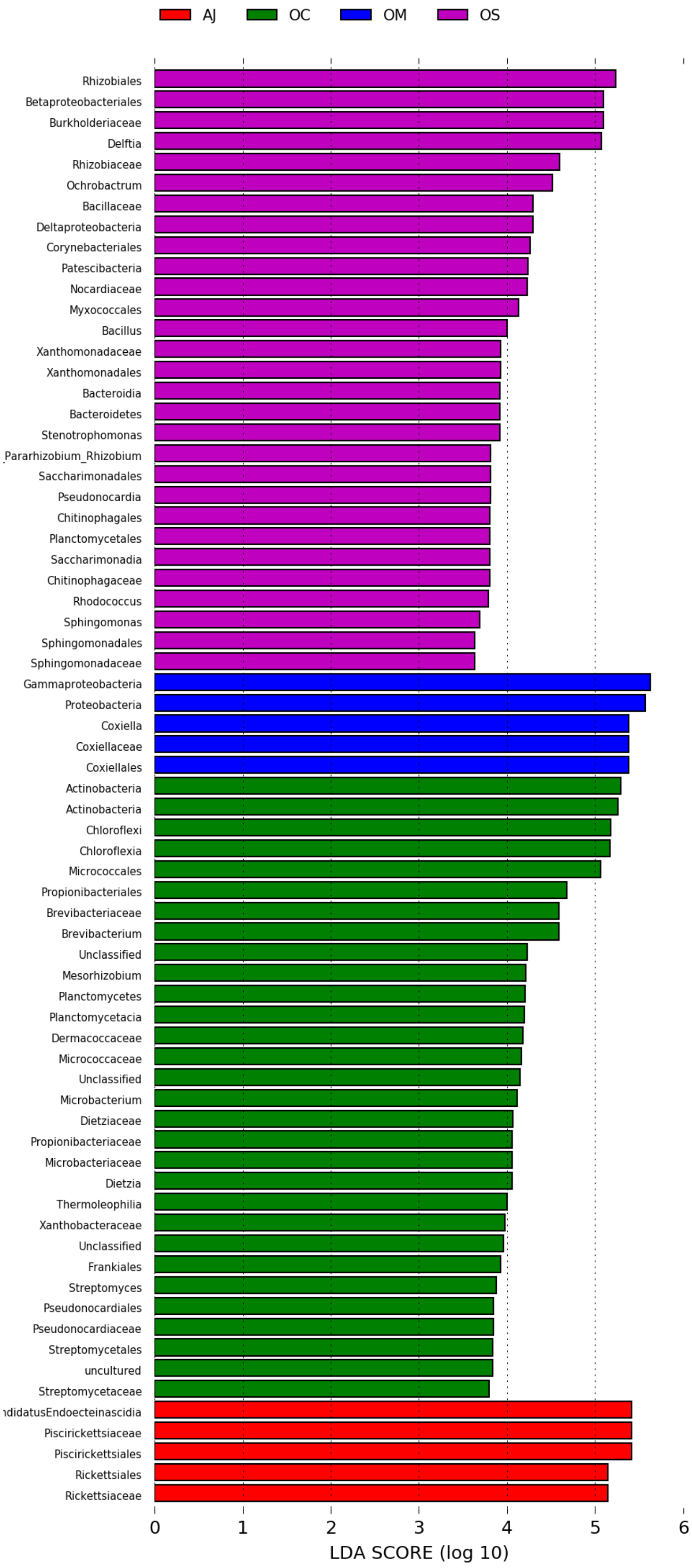

Fig. S16. LEfSe results showing the most differentially abundant taxa ($p < 0.05$) within male argasid ticks and color represents sample species (*A. japonicus* “AJ”, *O. capensis* “OC”, *O. moubata* “OM”, and *O. sawaii* “OS”).

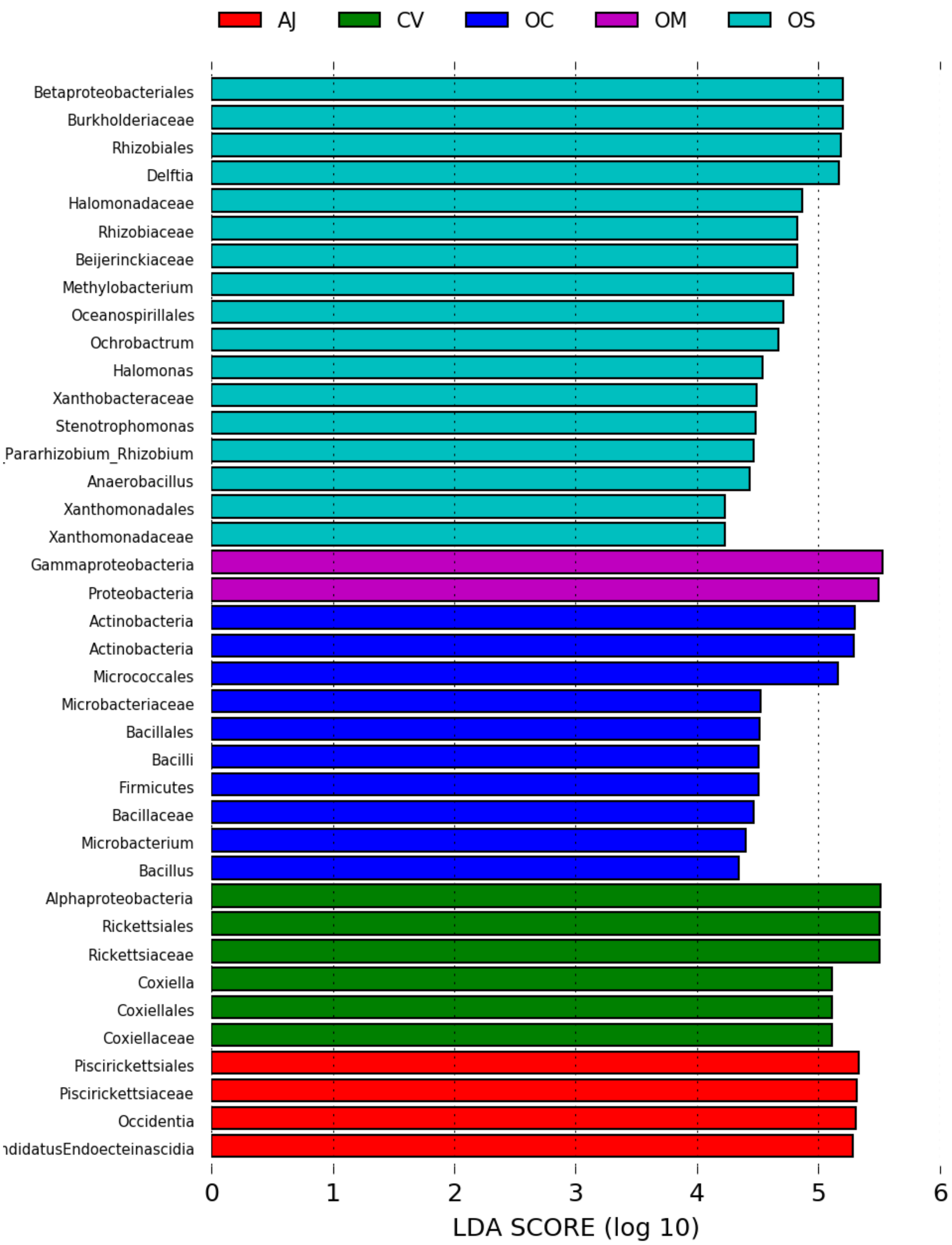

Fig. S17. LEfSe results showing the most differentially abundant taxa ($p < 0.05$) within nymph argasid ticks and color represents sample species (*A. japonicus* “AJ”, *C. vespertilionis* “CV”, *O. capensis* “OC”, *O. moubata* “OM”, and *O. sawaii* “OS”).

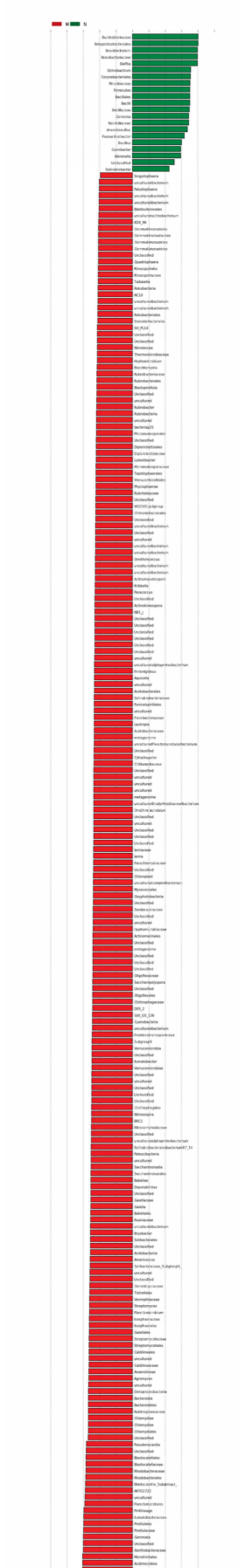

Fig. S18. LEfSe results showing the most differentially abundant taxa ($p < 0.05$) within male and nymph *O. capensis* samples.

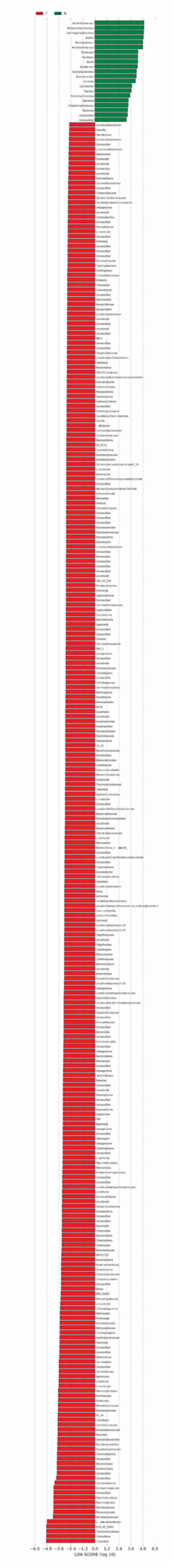

Fig. S19. LEfSe results showing the most differentially abundant taxa ($p < 0.05$) within female and nymph *O. capensis* samples.
